# Patient induced pluripotent stem cells identify specificities of a reticular pseudodrusen phenotype in age-related macular degeneration

**DOI:** 10.1186/s13073-026-01658-2

**Published:** 2026-05-10

**Authors:** Jenna C. Hall, Kavitha Krishna Sudhakar, Maciej Daniszewski, Anne Senabouth, Carla J. Abbott, Helena H. Liang, Himeesh Kumar, Grace E. Lidgerwood, Mehdi Mirzaei, Jessica YW Ma, Trevor Atkeson, Yumiko Hirokawa, Emeline F. Nandrot, Alexander Barnett, Chantal Cazevieille, Gaël Manes, Simon Mountford, Philip Thompson, Erica L. Fletcher, Zhichao Wu, Melanie Bahlo, Brendan R. E. Ansell, Daniel Paull, Alex W. Hewitt, Robyn H. Guymer, Joseph E. Powell, Alice Pébay

**Affiliations:** 1 Department of Anatomy and Physiology, The University of Melbourne, Parkville, VIC 3010 Australia; 2 Translational Genomics, Garvan Institute of Medical Research, Sydney, NSW 2010 Australia; 3 Centre for Eye Research Australia, Royal Victorian Eye and Ear Hospital, East Melbourne, VIC 3002 Australia; 4 Department of Surgery, Ophthalmology, The University of Melbourne, East Melbourne, VIC 3002 Australia; 5 Faculty of Medicine, Health and Human Sciences, Macquarie Medical School, Macquarie University, Sydney, NSW 2109 Australia; 6 Sorbonne Université, CNRS, INSERM, Institut de La Vision, Paris, 75012 France; 7 Menzies Institute for Medical Research, University of Tasmania, Hobart, TAS 7000 Australia; 8 Université de Montpellier, INSERM U1298, Institut Des Neurosciences de Montpellier, Hôpital Saint-Eloi, Montpellier, France; 9 Monash Institute of Pharmaceutical Sciences, Monash University, Parkville, VIC 3058 Australia; 10 Population Health and Immunity Division, Walter and Eliza Hall Institute of Medical Research, Parkville, VIC 3010 Australia; 11 Department of Medical Biology, The University of Melbourne, Parkville, VIC 3010 Australia; 12 The New York Stem Cell Foundation Research Institute, New York, USA; 13 School of Medicine, University of Tasmania, Hobart, TAS 7005 Australia; 14 CellTellus Laboratory, Melbourne, VIC 3000 Australia; 15 UNSW Cellular Genomics Futures Institute, University of New South Wales, Sydney, NSW 2052 Australia; 16 Department of Surgery, Royal Melbourne Hospital, The University of Melbourne, Parkville, VIC 3010 Australia

**Keywords:** Human induced pluripotent stem cells, Retinal pigment epithelium, Single-cell RNA sequencing, Genome regulation, Age-related macular degeneration, Reticular pseudodrusen, Transcriptomics, Proteomics

## Abstract

**Background:**

Age-related macular degeneration (AMD) is a leading cause of vision loss. Reticular pseudodrusen (RPD), deposits on the apical side of the retinal pigment epithelium (RPE), signify a distinctive and critical AMD phenotype. Yet, their molecular basis and relationship to the conventional drusen seen in AMD remain unclear.

**Methods:**

We generated induced pluripotent stem cell-derived RPE cells from a clinically phenotyped cohort comprising only individuals with conventional drusen (AMD/RPD-) or with drusen coexisting with RPD (AMD/RPD +). To identify differences between the two cohorts, we performed single-cell transcriptomic, proteomic, quantitative trait locus (QTL) and transcriptome-wide association (TWAS) analyses, together with functional assays.

**Results:**

AMD/RPD + RPE cells exhibited enrichment of extracellular matrix (ECM) and hypoxia-responsive pathways, and a relative underrepresentation of mitochondrial and oxidative phosphorylation processes, when compared with AMD/RPD- cells. Genetic analyses supported shared modulation of mitochondrial pathways across AMD, with additional regulatory signals associated with RPD risk. Functionally, all RPE cohorts formed drusen-like deposits in vitro. AMD/RPD- lines generated more basal deposits, whereas AMD/RPD + cells exhibited increased susceptibility to monolayer disruption.

**Conclusions:**

These findings indicate that AMD with and without RPD represent mechanistically distinct entities and provide novel insight into the molecular mechanisms underlying disease heterogeneity in AMD.

**Supplementary Information:**

The online version contains supplementary material available at 10.1186/s13073-026-01658-2.

## Background

Age-related macular degeneration (AMD) is a progressive, vision-threatening disease associated with dysfunction and death of the retinal pigment epithelium (RPE) and photoreceptors [1]. A pathological hallmark of AMD is the accumulation of drusen deposits underneath the RPE [2]. The increasing size of drusen is significantly associated with a higher risk of progression to late-stage disease [3]. However, AMD patients can present with drusenoid deposits on the apical side of the RPE in the subretinal space, called reticular pseudodrusen (RPD) or subretinal drusenoid deposits [4]. The presence of RPD in patients with AMD has been reported to be a significant risk factor for progression to late-stage AMD [5–7], which includes geographic atrophy and choroidal neovascularisation [8, 9]. The presence of RPD in individuals with AMD is also associated with significant visual function impairment [10, 11], especially in dark adaptation [12, 13]. Furthermore, there is evidence of treatment effect modification in the AMD/RPD + phenotype in a randomized controlled clinical trial for AMD, suggesting that different treatments may be required for these phenotypes of AMD [14]. The molecular composition of RPD shares some similarities with that of conventional drusen; however, significant differences in lipid content have been observed [15, 16], suggesting that distinct pathways are involved in the accumulation of conventional drusen and RPD [17].

It is hypothesised that both genetic and environmental factors contribute to RPD development. Elucidation of contributing factors has been hindered by difficulties in detection and by inconsistent criteria for identifying RPD presence (number of lesions and type of imaging used), complicating comparisons between studies. Several studies have examined the relationship between AMD-associated genetic variants and RPD [18]. The *ARMS2* rs10490924 single nucleotide polymorphism (SNP) has been consistently reported as enriched in individuals with AMD and RPD compared to those AMD eyes without RPD [19–27]. Associations with the *CFH* rs1061170 SNP have also been described [19, 21, 23, 24, 26, 27], although other studies failed to detect such a relationship [20, 22, 25]. Additional reported associations include variants in *C3* (rs22130199) [24], *C2/CFB* (rs641153) [24], *VEGFA* (rs943080) [24], *LIPC* (rs10468017) [27] and additional *CFH* SNPs (rs12144939, rs800292, rs393955, rs2274700) [23, 24]. However, many earlier studies used colour fundus photography and incident-case designs, which limited reliable RPD identification and made it difficult to distinguish RPD-specific from general AMD genetic effects. As these studies largely focused on known AMD variants and lacked RPD-specific stratification [10], genome-wide approaches in well-characterised AMD cohorts are needed to clarify RPD-specific genetic risk factors. Recent genome-wide association studies (GWAS) have confirmed the association of *ARMS2/HTRA1* rs11200638 SNP with RPD and also identified new genetic variants associated with RPD, rs79641866/*PARD3B*, rs143184903/*ITPR1*, and rs76377757/*SLN* [28] and a long non-coding RNA gene *HTRA1-AS1* (ENSG00000285955/BX842242.1) [29].

As induced pluripotent stem cells (iPSCs) [30, 31] can be differentiated into homogeneous RPE cultures, and have been widely used for disease modelling, including for AMD [32–41], we modelled RPE biology in vitro using patient iPSC-derived RPE cells. These cultures were from carefully phenotyped AMD donors, and categorised as either with (AMD/RPD +) or without RPD (AMD/RPD-).

## Methods

### Participant recruitment

All participants were over 50 years old and gave informed written consent. This study was approved by the Human Research Ethics committees of the Royal Victorian Eye and Ear Hospital (20/1459H; 11/1031H) as per the requirements of the National Health and Medical Research Council (NHMRC), in accordance with the Declarations of Helsinki and with the International Conference on Harmonisation guidelines for Good Clinical Practice. Cases were recruited through ongoing AMD natural history studies at the Centre for Eye Research Australia as follows: 1) AMD with only conventional drusen (AMD/RPD-, 56 individuals, 42 females, mean ± SD age at recruitment: 70.8 ± 7.9 years); and 2) AMD cases with both conventional drusen and with extensive RPD (AMD/RPD +, 57 individuals, 35 females, mean ± SD age at recruitment 78.6 ± 6.0). To determine the diagnoses of AMD and RPD, multimodal imaging was performed (Spectralis HRA + OCT, Heidelberg Engineering, Germany; Canon CR6-45NM, Canon, Japan) and graded by a senior retinal specialist (RHG). Optical coherence tomography volume scans were centred on the macula and comprised 49 horizontal B-scans (20° × 20°). The en-face modalities of colour fundus (50°), fundus autofluorescence (30°) and near-infrared imaging (30°) were also centred on the macula. All cases had to have imaging of sufficient quality to be able to grade for AMD stage and RPD status. Beckman classification was used for AMD staging [42]. Positive RPD phenotyping required at least five RPD lesions seen on two or more B-scans and confirmed on at least one en face modality. For this study, only eyes where RPD made up at least 50% of the total deposits were included in the AMD/RPD + cases (extensive RPD). Negative RPD phenotyping was defined as < 5 RPD lesions.

### Biopsy processing and fibroblast culture

Skin biopsies were obtained from non-sun-exposed regions using a 3 mm^2^ dermal punch, stored in cold biopsy collection medium (DMEM high glucose, 100 U/mL penicillin and 100 mg/mL streptomycin and 250 ng/L fungizone) and processed on the day of collection. Fibroblasts were expanded, cultured, and banked in DMEM high glucose, 10% fetal bovine serum, L-glutamine, 100 U/mL penicillin and 100 mg/mL streptomycin (all from Thermo Fisher Scientific, USA). All cell lines were mycoplasma-free. Fibroblasts at passage (p) 2 were used for reprogramming.

### Generation, selection, maintenance and quality control of iPSC lines

The reprogramming, maintenance and passaging of iPSCs were performed as described [43]. Participants’ iPSCs were generated by nucleofection (Neon™ Transfection System, Thermo Fisher Scientific) of episomal vectors using Epi5™ Episomal iPSC Reprogramming kit (Thermo Fisher Scientific) in feeder-free and serum-free conditions using TeSR™-E7™medium (Stem Cell Technologies) as we previously described [43]. Pluripotent cells were selected using anti-human TRA-1–60 Microbeads (Miltenyi) [44], maintained onto vitronectin XF™-coated plates (Stem Cell Technologies) in StemFlex™ (Thermo Fisher Scientific), with media changes every 2–3 days and weekly passaging using ReLeSR™ (Stem Cell Technologies). The iPSC lines from AMD/RPD + (MBE-03537, MBE-03393, MBE-03536, MBE-03579, and MBE-03589), AMD/RPD- (MBE-03556, MBE-03587, MBE-03590, MBE-03408, MBE-03409, TOB-01932) and healthy controls (WAB-00005, WAB-00014, WAB-00025, WAB-00033, WAB-00134, WAB-00546, TOB-01986) were previously generated and characterised in [40, 45, 46], respectively. Pluripotency was assessed by expression of OCT3/4 (sc-5279, 1:40, Santa Cruz Biotechnology) and TRA-1–60 (MA1-023-PE, 1:100, Thermo Fisher Scientific) by immunocytochemistry with nuclei counterstained using bisBenzimide Hoechst 33,342 trihydrochloride (#B2261, Sigma-Aldrich). Virtual karyotyping by copy number variant (CNV) analysis was performed as we previously described [43], using PennCNV and QuantiSNP with default parameter settings. Chromosomal aberrations were deemed to involve at least 20 contiguous SNPs or a genomic region spanning at least 1.5 Megabases.

### Differentiation of iPSCs into RPE cells

RPE cells were generated as described [47]. Briefly, iPSCs were plated on vitronectin (STEMCELL Technologies, 07180) diluted with CellAdhere buffer (STEMCELL Technologies #07183) at a final concentration of 10 µg/mL and hiPSCs were maintained in a 37 °C, 5% CO_2_ incubator with daily medium changes with Stemflex (Life Technologies, A3349401) until iPSC colony confluency reached 60%. StemFlex medium was replaced with RPE Progenitor Specification Medium, TeSR-E6 (STEMCELL Technologies, 05946) supplemented with N2 (ThermoFisher Scientific, 17,502,048) with media changes occurring every second day. After 30 days, the medium was switched to RPEM ((MEMα) (Life Technologies, 12,561,072) supplemented with 5% foetal bovine serum (FBS) (Life Technologies, 26,140,079), MEM NEAA (Life Technologies, 11,140–050, 0.1 mM), N1 supplement (Sigma Aldrich, N6530-5ML, 0.1 mM), 1% L-Glutamine–Penicillin–Streptomycin solution (Sigma Aldrich, G1146-100ML), taurine (Sigma Aldrich, T-0625, 250 µg/mL), hydrocortisone (Sigma Aldrich, H6909, 20 ng/mL) and triiodothyronine (Sigma Aldrich, T-5516, 100 ng/mL)). Cells were subsequently passaged with 0.25% trypsin–EDTA and plated onto growth factor-reduced Matrigel-coated plates (#354,230, Corning) at a final concentration of 0.035 mg/cm^2^ to enrich in RPE cells for a final 30 days (90 days total). To minimise experimental variation, all iPSC lines were differentiated into RPE cells across five independent differentiation batches, with each batch including multiple lines from both AMD/RPD + and AMD/RPD- cohorts.

### Immunocytochemistry

At Day 60, iPSC-derived RPE cells were plated onto Matrigel-coated 96-well CellCarrier Ultra plates (PerkinElmer, 6,055,300; 0.035 mg/cm^2^). After 30 days in culture with medium changes every other day, cells were fixed with 4% paraformaldehyde (10 min, room temperature) and immunostained using standard procedures with the following primary and secondary antibodies: ZO-1 (339,100, 10 μg/mL, Life Technologies), PMEL (ab137062, 5 μg/mL, Abcam), RPE65 (ab235950, 10 μg/mL, Abcam), CRALBP (MA1-813, 10 μg/mL, ThermoFisher), APOE (701241, 3 µg/mL, ThermoFisher Scientific), AlexaFluor 647 Phalloidin, AlexaFluor 488 goat anti-mouse IgG and AlexaFluor 568 goat anti-rabbit IgG (ThermoFisher Scientific, A22287, A11029, A11011 respectively). Nuclei were counterstained with Hoechst 33,342 (Sigma, B2261). Staining specificity was confirmed using appropriate isotype controls. Imaging was performed by full-plate scans (5 fields/well) at 20 × magnification on a PerkinElmer Operetta system.

### Transmission electron microscopy

RPE cells were dissociated using 0.25% Trypsin–EDTA (Life Technologies, 25,200,072), pelleted (5 min, 300 g), and fixed in 2.5% EM-grade glutaraldehyde (Electron Microscopy Sciences, 111–30-8) in 1 × sodium cacodylate buffer (Sigma, C0250) for 2 h at room temperature, then stored at 4 °C. After 24 h, cells were washed, resuspended in 0.5% glutaraldehyde, and maintained at 4 °C until shipment. On the day of shipment, cells were pelleted, resuspended in cacodylate buffer, and shipped at ambient temperature to the Institut des Neurosciences de Montpellier (France) for processing and imaging. Upon receipt, cells were immersed in 2.5% glutaraldehyde in PHEM buffer (pH 7.4) overnight at 4 °C, rinsed in PHEM, and post-fixed with 0.5% osmium tetroxide and 0.8% potassium hexacyanoferrate trihydrate for 2 h at room temperature in the dark. Samples were dehydrated through a graded ethanol series (30–100%) and embedded in EmBed 812 using a Leica EM AMW microwave tissue processor. Ultrathin sections (70 nm) were cut using a Leica-Reichert Ultracut E, stained with 1.5% uranyl acetate in 70% ethanol and lead citrate, and imaged using a Tecnai F20 transmission electron microscope at 120 kV (INM, Université de Montpellier, INSERM U1298).

### Transepithelial electrical resistance assay

The electrodes of the epithelial voltohmmeter (EVOM) were first rinsed with 70% ethanol. The EVOM was normalised and a blank measurement was recorded by submerging the electrodes in RPEM. All transepithelial electrical resistance measurements were made under sterile conditions in a cell culture hood, and each sample was measured with the basolateral probe touching the well bottom and with the chopstick electrode at the same angle of approach to avoid significant variability in transepithelial electrical resistance measurements. Net transepithelial electrical resistance measurements were calculated by subtracting the blank value (Matrigel-coated filter without cells) from the experimental value. Each reading was performed in duplicate from two independent experiments. Final resistance-area products (Ω·cm^2^) were obtained by multiplying by the effective growth area.

### Photoreceptor outer segment harvest and assay

Photoreceptor outer segments (POS) were purified from fresh porcine eyes as described previously [48]. Briefly, full retinas were collected under dim red light in a homogenizing solution (20% sucrose, 20 mM tris acetate pH7.2, 2 mM MgCl_2_, 10 mM glucose, 5 mM taurine) and shaken thoroughly. Retinal homogenates were filtered and deposited onto continuous 25–60% sucrose gradients (tris acetate pH7.2, 10 mM glucose, 5 mM taurine). After ultracentrifugation (50 min, 25,000 rpm, 4 °C), the orange band containing POS was collected and diluted 1:4 in wash solution 1 (20 mM tris acetate pH7.2, 5 mM taurine). POS were then centrifuged (10 min, 5,000 rpm, 4 °C), washed further in solutions 2 and 3 (10% sucrose, 20 mM tris acetate pH7.2, 5 mM taurine; 10% sucrose, 20 mM Na phosphate pH7.2, 5 mM taurine), with centrifugation steps in between each wash. POS were labeled using 1 mg/mL fluorescein isothiocyanate in DMEM (90 min, RT). POS were washed twice in wash solution 3 and once in DMEM. After a last resuspension in DMEM, POS were counted, aliquoted after addition of sucrose to a final concentration of 2.5% and stored at −80 °C. Phagocytosis was assessed by flow cytometry following incubation of RPE cells with FITC-labelled POS (FITC-POS). Freshly thawed FITC-POS were diluted into CO_2_-independent medium (ThermoFisher, #18,045,088) and incubated with RPE cells (200,000 FITC-POS/cm^2^) for 3.5 h at 37 °C. RPE cells were washed twice with filtered PBS^+/+^ (Life Technologies, 14,040–133) to remove unbound POS, then RPE were dissociated using trypsin–EDTA (0.25%), phenol red (Life Technologies, 25,200,072) at 37 °C to dislodge cells and remove bound POS from the cell surface. Trypsin was inactivated with RPEM, cells were triturated to generate a single cell suspension containing RPE cells and bound FITC-POS. RPE cells were incubated with DAPI (0.1 µg/mL, Miltenyi, 130–111–570), passed through a 35 µm cell strainer, then analysed on a Becton Dickinson FACS Aria III flow cytometer. Analysis was performed using FCS Express 5 (DeNovo Software). The percentage of RPE cells that internalised FITC-POS was measured from the DAPI-negative gated live cell population. This population was initially gated by size (Forward vs Side scatter) to exclude unbound POS and any cell debris.

### Drusen-like deposit quantification

Confocal imaging was performed using a Zeiss LSM 900 confocal microscope equipped with 2 fluorescence GaAsP, 1 Airyscan detector and transmitted light ESID detector along with 4 diode lasers (405, 488, 561 and 640 nm). The 405, 488, and 561 nm lasers and transmitted light were used in each imaging session. Images were acquired from samples fixed in a 96-well Cell Carrier Ultra Plate in immersion media PBS^−/−^ (Life Technologies, 14,190–144) using a 20x/0.8 NA Air Objective. Upon instrument initialization, the plate was calibrated in the Zen 3.2 software and three random points for unbiased imaging were distributed across each well. It was common for the thickness across samples to be highly variable, and it was thus essential that the final Z-stack range was set to accommodate the thickest sample. Automated Z-stack centering was deployed using autofocus during the scan. ZO-1 (excited with 488 nm), APOE (excited with 561 nm), Hoechst (excited with 405 nm) and brightfield (transmitted light) channels were imaged with a Z-stack interval of 0.54 µm over a range of 50 µm (~ 100 sections). The output of the semi-automated scan was three 50 μm Z-stacks (.czi files) per well. Imaris (v9.9, Oxford Instruments) was used for processing the Z-stacks acquired during the Zeiss LSM 900 overnight scan. The Zen image files (.czi) were transferred to the Imaris arena and converted to an Imaris file format (.ims) using an Imaris File Converter (v9.9.1). Using the optimised image processing and quantification parameters described below, the software renders a 3D version of the Z-stack and volume-fills each dye. This allowed for quantification of the amount and volume of the drusen-like deposits, measurements that were then exported to.csv file for further statistics. Using Imaris batch analysis, conditions were optimised and individual images were modified in the following workflows: Baseline Subtraction—the background value to be subtracted for each channel was determined using a region selection tool to randomly select three background areas to measure the mean intensity in FIJI. The average of these three measurements was used as the background value with grey values of 6666 and 1111 measured for the APOE (Alexa Fluor 568) channel and ZO-1 (Alexa Fluor 488) respectively. The Baseline Subtraction tool in Imaris was then used to subtract this baseline value from the intensity of every voxel in the image for the APOE and ZO-1 channels. Median Filter—a median filter (3 × 3 × 1) was applied to the Hoechst 33,342 channel to reduce background intensity while preserving the edges of the nuclear structure. Surfaces—image segmentation to identify nuclei and drusen-like deposits was performed using the Surfaces tool in Imaris. This tool was used to render a 3D object representing the fluorescent volume in the Z-stack of each channel. The resulting slices were used to aid in visualisation alongside quantitative analysis including spatial and intensity statistics such as deposit count, cell count, and volume of the deposits.

### N-Retinylidene-N-Retinylethanolamine synthesis and treatment

N-Retinylidene-N-Retinylethanolamine (A2E) was synthesised following the protocol in [49]. A2E treatment was carried out in RPE cells cultivated in 96-well Cell Carrier Ultra plates matured for 6 months in RPEM . A2E concentration and treatment schedule (10 µm, every other day, in the dark, for 7 days) were established based on [50].

### Cell detachment analysis

RPE monolayers were visually inspected under phase-contrast microscopy. Detachment was defined as partial or complete loss of the adherent RPE monolayer from the culture surface, evident as visible peeling, lifting, or absence of contiguous cell coverage across substantial areas of the well. For each line, the presence or absence of detachment was recorded per well. The proportion of detached wells was calculated separately for control, AMD/RPD-, and AMD/RPD + cohorts.

### RPE cell harvest and single-cell preparation

RPE cells were dissociated using trypsin–EDTA (0.25%) in phenol red (Life Technologies, 25,200,072) for 5 min at 37 °C in a 5% CO_2_ incubator, the plate was tapped to release any non-RPE cells and washed with PBS^−/−^. Trypsin–EDTA (0.25%) in phenol red was added a second time and incubated for 10 min at 37 °C in a 5% CO_2_ incubator. The trypsin was inactivated with RPE medium and cells were then harvested into a 15 mL conical tube, as a single-cell suspension. The cell suspension was centrifuged (5 min, 300 g, 4 °C), then resuspended in 1 mL of 0.1% BSA-PBS. Cells were counted and assessed for viability with trypan blue, and pooled (eight samples maximum) at a concentration of 1000 live cells/μL (1 × 10^6^ cells/mL). The pooled samples were combined with the master mix containing the reverse transcriptase reaction and added to the first well of the third row of the 10 × Genomics Chromium Test Chip. Partition oil was loaded into the first well of the first row, and gel beads were added to the first well in the second row. The remaining wells were filled with 50% glycerol and the bottom row was empty. As the chip runs, an emulsion occurs wherein each oil droplet contains one bead and one single cell termed GEM.

### Single cell 3’ RNA-sequencing and pre-processing of transcriptome data

Single cell RNA-sequencing libraries were prepped using the Chromium Single Cell 3’ Library & Gel bead Kit for 10X Genomics. Libraries were sequenced on Illumina NovaSeq 6000. Raw base calls (BCL files) from the sequencer were demultiplexed into FASTQ format using the *bcl2fastq* software (https://sapac.support.illumina.com/sequencing/sequencing_software/bcl2fastq-conversion-software.html). Read quality control, alignment, cell and unique molecular identifier (UMI) counting was performed using Cell Ranger *count* pipeline from 10X Genomics (v6.0.2 was run on the first batch and v7.1.0 on the remaining three batches) with the default target cell count at 20,000 and Homo Sapiens GRCh38 (GRCh38-2020-A) as the reference. Quality control filters were tailored to each library to account for differences in sequencing depth. The aim was to remove the lower and upper outlier barcodes. Additionally, barcodes that could not be confidently assigned to a donor or were deemed a doublet were also excluded (see the *Demultiplexing of cell pools into individual donors* section for details). The mitochondrial gene expression threshold was set to 30% of the total transcripts. Normalisation and scaling were performed using *Seurat’s* SCTransform method with ribosomal gene content percentage regressed out. All libraries were then merged and integrated using *Harmony* R package (version 1.2.0) with the sequencing pool as a covariate. Sample variables like sex and age were tested and were not found to cause batch effects.

### SNP genotyping and imputation

DNA was extracted from cell pellets using QIAamp DNA Mini Kit (QIAGEN, 51,306) as per the manufacturer's instructions. DNA concentration was determined with a SimpliNano spectrophotometer (GE Life Sciences), and DNA was diluted to a final concentration of 10–15 ng/µl. Samples were genotyped on the UK Biobank Axiom™ Arrays (Ramaciotti Centre for Genomics, Sydney, Australia). Pre-processing of the raw CEL files and VCF generation was done using Axiom Analysis Suite (version 5.3.0.45) from Thermo Fisher Scientific. Genotype imputation was run using the SNP_imputation_1000g_hg38 pipeline using the 1000G Project as the reference (https://github.com/powellgenomicslab/SNP_imputation_1000g_hg38/tree/master). Imputation quality filters were set to minor allele frequency of > 10%, R^2^ score of 0.8 and fraction of missing genotypes of 0.5, resulting in 4,063,692 SNPs.

### Demultiplexing of cell pools into individual donors

Demultiplexing and doublet detection for the libraries was run with *Demuxafy* (version 3.0.0) using the SNP genotyping data as reference (https://demultiplexing-doublet-detecting-docs.readthedocs.io/en/latest/Background.html). The softwares chosen for demultiplexing were *demuxlet*, *demuxalot* and *souporcell*, while *scds* and *scDblFinder* were used for doublet detection. The parameters for all methods were set to the defaults in *Demuxafy*. The results were consolidated using the “MajoritySinglet” approach in combine_results.R of *Demuxafy*. The final assignments were incorporated into the Seurat objects.

### Classification of cell subpopulations

The first 20 principal components from Harmony embedding were chosen for the Uniform Manifold Approximation and Projection (UMAP) visualisation. The optimal clustering resolution was deduced as 0.3 using the gap statistic method from scBubbleTree which resulted in 14 clusters. Canonical marker genes associated with RPE and proliferative functions were examined for each cluster, as we previously described [40]. As a first pass, we performed label transfer using the previously described RPE cells as reference [38]. Additionally, markers for each of the clusters were also investigated using Seurat’s *FindMarkers* function. Clusters that undoubtedly demonstrated proliferative markers were labelled as RPE progenitors. The remaining clusters showed varying levels of RPE functions and were deduced as either early RPE or RPE.

### Differential expression analysis

Since the main focus of this study was understanding the variance within disease phenotypes rather than specific states of maturity, we combined all RPE subpopulations (early and mature RPE) into a single group and interrogated differences between the two disease phenotypes within this combined group. This helped increase the power of the calculations. Moreover, in some of the smaller RPE populations, not all donors were represented. The analysis was performed using the MAST (v1.28.0) implementation within the Seurat package using log-transformed normalised counts from SCTransform. Covariates included as latent variables include total UMI count, sequencing pool, sex and age. Thresholds for significant results were set to | Average log2 Fold Change |> 0.25 and adjusted *p* value < 0.05.

### Gene ontology and disease ontology overrepresentation analysis

For each disease phenotype, gene sets for overrepresentation analysis were prepared using the significant hits from differential expression analysis. Overrepresentation analysis was performed with the *clusterProfiler* (v4.10.1) package [51, 52] to identify enriched pathways and processes using three resources: Gene Ontology, Disease Ontology (DOSE v3.28.2) and KEGG pathways. The *P* value threshold was set to < 0.05 and false discovery rate of < 0.05.

### Mapping of expression and protein QTL

To explore any association between genetic variation and gene expression in the context of disease states, we performed eQTL analysis using a pseudobulk approach with *matrixEQTL* (v2.3) (https://github.com/andreyshabalin/MatrixEQTL). For each population, an aggregated donor-gene matrix was generated by running quantile normalisation on the mean values of each gene. The analysis was run using an additive linear model on all non-zero genes and 4,063,692 SNPs. For each gene, SNPs located within a 1 MB window from start and end of the gene were tested. Covariates specified were age, sex, top five genotype principal components and disease state. Significant eQTLs were filtered with the following thresholds: false discovery rate < 0.05 and homozygous alternate allele frequency of at least 5. Within the subset of significant eQTLs, to test whether the effect of the eQTLs differed between the disease states, we added a Genotype:Phenotype interaction term to the model and filtered by *P* value < 0.05 of the interaction term.

pQTL analysis was run on bulk-level protein abundance measurements from 69 RPE cell lines. The abundance matrix was normalised using rank-based inverse transformation. Genotype data of the RPE cell lines gave 4,078,773 SNPs (minor allele frequency > 10%) after QC. Covariates incorporated into the model were age, sex, top 10 genotype principal components and disease state. Association test for each protein was run with *QTLtools cis* permutation test (v1.3.1) [53] using SNPs within a 1 MB window. False discovery rate values were calculated on the variant-level adjusted empirical *P*-values and filtered by < 0.05 for significant pQTLs.

### Transcriptome wide association study

Transcriptome wide association study (TWAS) analysis was performed with the FUSION package [54] with the following inputs: a) gene expression weights calculated with *FUSION.compute_weights.R* using matrixEQTL summary statistics with “blup”, “lasso”, “top1” and “lasso” models and, b) RPD-specific GWAS summary statistics [29]. The publicly available 1000 Genomes LD reference file (https://github.com/gabraham/plink2R/archive/master.zip) was used since we did not have enough samples to create our own. False discovery rate values were calculated on the association *P* values for each cell type then filtered by 10% for significance.

### Preparation of protein samples

RPE cell cultures were lysed in RIPA buffer containing phosphatase and protease inhibitors, then sonicated (40 Hz, 2 pulses of 15 s each) using a probe sonicator. Insoluble debris was removed by centrifugation at 14,000 rpm for 15 min at 4 °C. Protein concentrations were measured using the bicinchoninic acid protein assay kit (Thermo Scientific). Extracted proteins (50 μg) were adjusted to 50 μL in 1 × S-Trap lysis buffer and reduced/alkylated with TCEP and MMTS. The S-Trap midi protocol (Protifi) was performed. Phosphoric acid was added to the SDS lysate (final concentration 1.2%), followed by the addition of 350 μL S-Trap binding buffer (90% aqueous methanol with 100 mM TEAB). The mixture was loaded onto an S-Trap column, washed, and digested with trypsin at a 10:1 protein ratio in 50 mM TEAB. After overnight incubation at 37 °C, peptides were eluted in three stages, pooled, and concentrated using a Speed-Vac to near dryness (~ 5 μL remaining).

### Tandem mass tag labelling and peptide fractionation

Proteome profiling was conducted on a Tandem Mass Tag (TMT) platform with six independent 16-plex TMT experiments. Dried peptides were resuspended in 100 mM HEPES buffer (pH 8.2), and concentrations were determined with the MicroBCA kit. Peptides from each sample (35 μg) were labelled with 0.2 mg TMT reagent per tube for 1 h at room temperature with vortexing. Residual TMT reagent was quenched with 8 μL of 5% hydroxylamine. For each 16-plex experiment, labelled samples were combined and dried by vacuum centrifugation. The peptides were cleaned using a C18 column (Sep-pak, Waters) before undergoing high-pH reversed-phase fractionation on an Agilent 1260 HPLC system. Peptides were separated using a 55-min gradient from 3–30% acetonitrile in 5 mM ammonia (pH 10.5) at a flow rate of 0.3 mL/min. The collected 96 fractions were consolidated into eight final fractions. These were dried, resuspended in 1% formic acid, and desalted using SDB-RPS stage tips.

### Liquid chromatography and tandem mass spectrometry

Mass spectrometric data were acquired using an Orbitrap Eclipse mass spectrometer connected to a Vanquish Neo UHPLC system. Approximately 1 µg of peptide was separated on a 100 µm capillary column packed with 35 cm of Accucore 150 resin (2.6 μm, 150 Å; ThermoFisher Scientific) at a flow rate of 350 nL/min. The scan sequence started with an MS1 spectrum (Orbitrap analysis, resolution set to 60,000, mass range 350–1350 Th, automatic gain control target set to 100%, maximum injection time set to “auto”. Data acquisition lasted 75 min per fraction. The high-resolution MS2 (hrMS2) stage involved fragmentation via higher-energy collisional dissociation at a normalized collision energy of 36%. Analysis was performed using the Orbitrap (automatic gain control target 200%, maximum injection time 86 ms, isolation window 0.5 Th, resolution 30,000, with TurboTMT enabled). The FAIMSpro interface was used, with a dispersion voltage of 5,000 V and compensation voltages set at + 30 V, −50 V, and −70 V. The TopSpeed parameter was set to 1 s per compensation voltages.

### Proteomic data analysis

Spectra were converted to mzXML format using MSconvert [55]. Database searches were conducted using all entries from the homo sapiens UniProt reference database (downloaded: June 2024) and a reversed concatenated sequence database. Searches were performed using a 50-ppm precursor ion tolerance for protein profiling and a product ion tolerance of 0.03 Da, maximizing sensitivity for Comet searches and linear discriminant analysis [56, 57]. Static modifications included TMTpro labels on lysine residues and peptide N-termini (+ 304.207 Da), along with carbamidomethylation of cysteine residues (+ 57.021 Da). Oxidation of methionine residues (+ 15.995 Da) was set as a variable modification. Peptide-spectrum matches (PSMs) were adjusted to a 1% false discovery rate [58, 59]. PSM filtering utilized linear discriminant analysis [57, 59], and proteins were assembled to a final protein-level false discovery rate of 1% [59]. Protein quantification was based on summing reporter ion counts from all matching PSMs [60]. Reporter ion intensities were corrected for isotopic impurities according to manufacturer specifications. The signal-to-noise measurements for peptides assigned to each protein were summed, and these values were normalized to ensure equal protein loading across channels. Each protein abundance was then scaled such that the summed signal-to-noise ratio for that protein across all channels equaled 100, resulting in a relative abundance (RA) measurement. Differential expressed proteins were identified through Student’s *t*-tests, specifically comparing the ratios between both groups. The overall fold changes were calculated as geometric means of the corresponding ratios. For a protein to be considered differentially expressed, we followed the following two criteria: a ratio fold change exceeding 1.2 for upregulated or falling below 0.83 for downregulated expression, and a *p* value cutoff (*t*-test *p <* 0.05). Investigation of protein–protein interactions and functional enrichment gene ontology analysis of differentially expressed proteins were performed with the online STRING database version 12.0119. STRING analysis on the 715 differentials expressed proteins within the proteomics dataset, generated a network of interactions (based on both evidence of functional and physical interactions). The top hits were selected based on the minimum required interaction score of 0.9 (highest confidence), and the full STRING network was deployed to visualise network maps and interactors. Network lines represent the protein interaction score, which was set at a minimum high confidence (0.7) for visualisation. Active interaction sources were based on text mining, experiments, databases, coexpression, neighbourhood, gene fusion, and co-occurrence data. The network analysis uses a strength score of each STRING network, which is the ratio between the number of proteins in the specified network that are annotated with a term, and the number of proteins that STRING expects to be annotated with this term in a random network of the same size (Log10(observed/expected) = strength).

## Results

### Generation of iPSCs, differentiation into RPE cells, and genomic profiling

Fibroblast cultures generated from skin biopsies of 103 individuals exhibiting either only drusen (AMD/RPD-) or drusen coexisting with extensive RPD (AMD/RPD +) were reprogrammed into iPSCs (72 females and 31 males; mean ± SD: 74.6 ± 7.9 years at recruitment) (Additional file 1: Fig. S1). The iPSC lines were genotyped for 845,487 SNPs and imputed with the Haplotype Reference Consortium panel [61]. Following quality control assessments, a total of 4,063,692 autosomal SNPs were obtained with minor allele frequency > 10%. To minimise experimental variation, all lines were differentiated into RPE cells in five independent batches as previously described [40], and suboptimally differentiated cultures (as assessed by homogeneous cobblestone morphology and pigmentation) were excluded from analysis. This method yields functional RPE cells that express canonical RPE cell markers (Additional file 1: Fig. S1). Differentiated cell lines were divided into 13 pools, each consisting of up to 8 cell lines from both groups. scRNA-seq was performed on all pools, targeting 20,000 cells per pool and sequencing at 30,000 reads per cell. The resulting single-cell transcriptome profiles underwent quality control and donor assignment to remove cells designated as doublets or from individual lines with a low number of assignment anomalies identified by CNV array (10 lines) and that failed genotyping. By the end of quality control assessment, 130,524 cells from 68 individual lines remained: 35 lines from participants with conventional AMD only (AMD/RPD-, 66,882 cells, seven males, 28 females, mean ± SD age: 71.1 ± 8.5 years) and 33 lines from participants with RPD (AMD/RPD +, 63,642 cells, 14 males, 19 females, mean ± SD age: 79.7 ± 1.0 years).

**Fig. 1:**
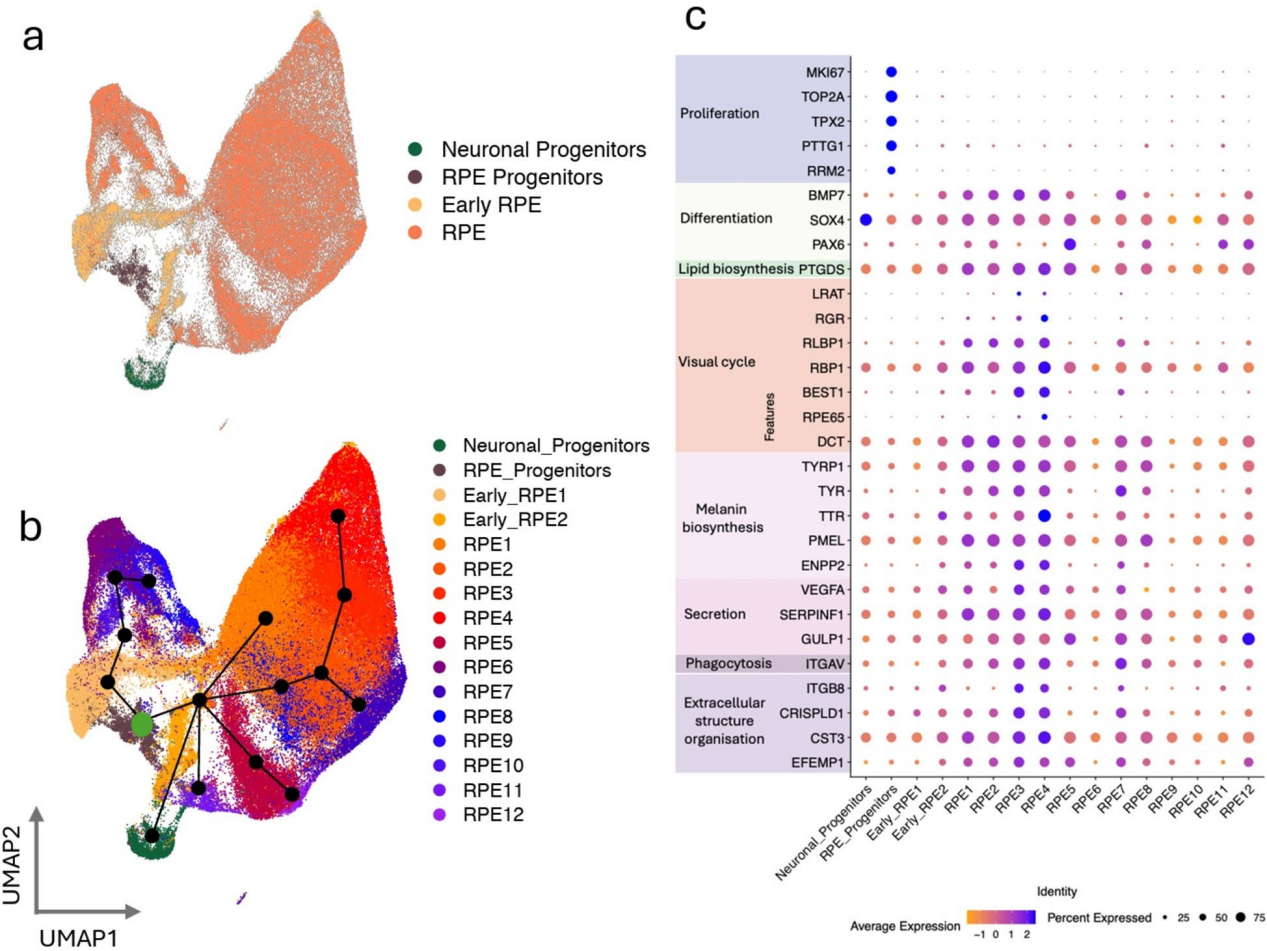
Characterisation of subpopulations. **a** Uniform Manifold Approximation and Projection (UMAP) of cells labeled by subpopulation identity and (**b**) showing cell trajectory. **c** Dotplot showing scaled average expression (z-scores; colour scale) and percentage of expressing cells (dot size) for genes associated with RPE functions (extracellular structure organization, phagocytosis, secretion, melanin biosynthesis, visual cycle, lipid biosynthesis, differentiation, and proliferation) across subpopulations

The cells were distributed among 16 subpopulations which were detected in both subphenotypes at comparable frequencies (Table 1), and classified across subtypes of neuronal progenitors, RPE progenitors and RPE cells. Consistent with previous observations [40], transcriptomic differences among the 14 RPE subpopulations primarily reflect changes in maturity, rather than in cell identity (Fig. 1). For downstream analyses, these RPE clusters were therefore aggregated. Genes associated with AMD were expressed across all subpopulations in both cohorts (Additional file 1: Fig. S2). Of note, no significant difference in *CFH* and *C3* were observed between cohorts (Additional file 1: Fig. S2).

**Table 1:** Number and % of cells per subphenotypes and subpopulations

Subpopulation	AMD/ RPD-	AMD/ RPD +	Total	% AMD/ RPD-	% AMD/ RPD +
Early RPE1	6,476	4,755	11,231	9.68	7.47
Early RPE2	3,649	2,679	6,328	5.46	4.21
Neuronal Progenitors	1,549	1,273	2,822	2.32	2.00
RPE Progenitors	2,081	1,661	3,742	3.11	2.61
RPE1	10,600	11,223	21,823	15.85	17.63
RPE2	8,756	8,450	17,206	13.09	13.28
RPE3	7,324	6,594	13,918	10.95	10.36
RPE4	6,185	6,026	12,211	9.25	9.47
RPE5	6,225	3,997	10,222	9.31	6.28
RPE6	3,977	5,695	9,672	5.95	8.95
RPE7	3,504	3,407	6,911	5.24	5.35
RPE8	3,220	2,793	6,013	4.81	4.39
RPE9	1,094	2,915	4,009	1.64	4.58
RPE10	600	1,198	1,798	0.90	1.88
RPE11	1,085	658	1,743	1.62	1.03
RPE12	557	318	875	0.83	0.50
Total	66,882	63,642	130,524	100	100

### Differential gene expression between AMD*/RPD* + and AMD/RPD- RPE

**Fig. 2:**
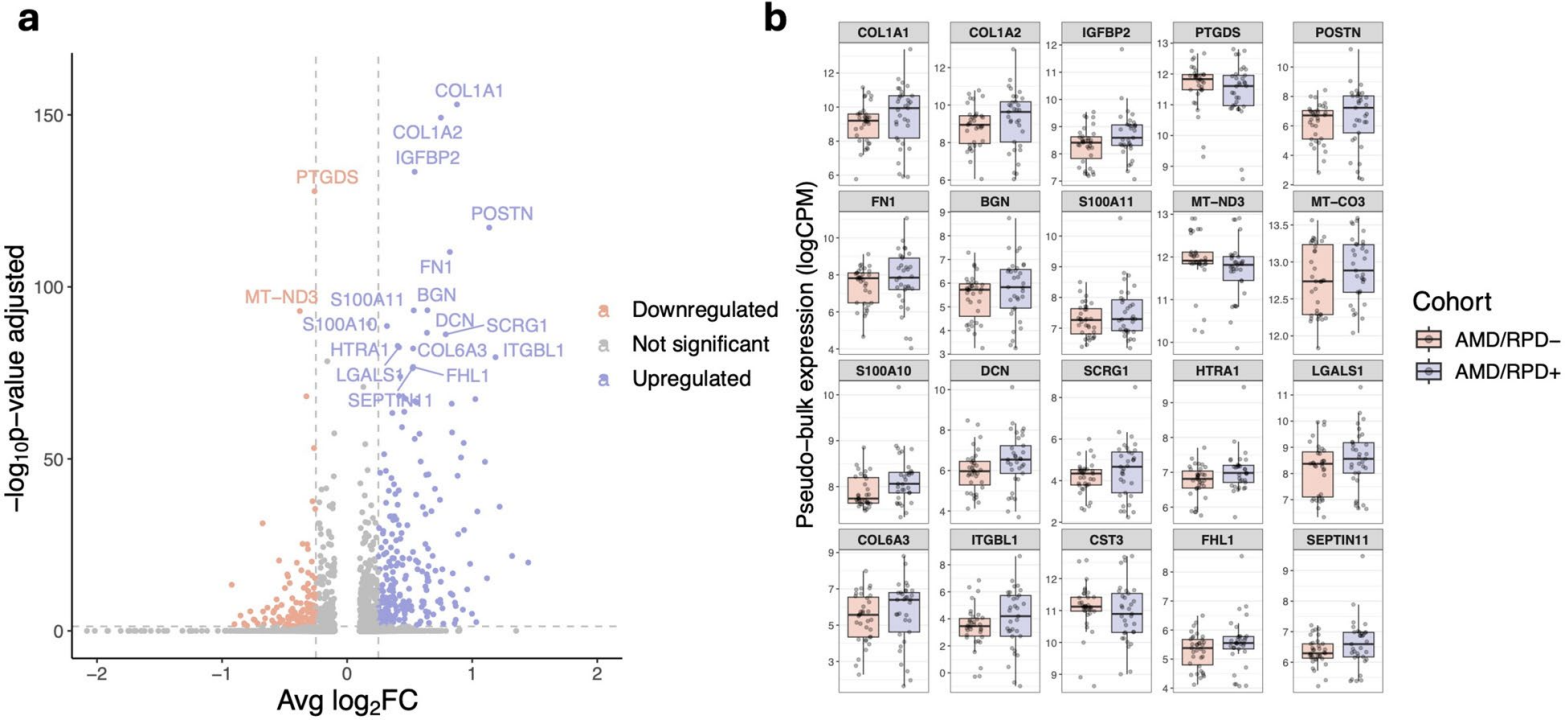
Differential gene expression in RPE cells from AMD/RPD + and AMD/RPD- participants. **a** Volcano plot showing differentially expressed genes between RPE cells derived from AMD/RPD + and AMD/RPD- participants (directionality relative to AMD/RPD +). Genes significantly downregulated in AMD/RPD + (adjusted *p <* 0.05, log₂FC > 0.25) are shown in red, and upregulated genes in blue. The top 20 differentially expressed genes are annotated. **b** Box plot of top 20 genes showing pseudobulk expression in logCPM (counts per million) across AMD/RPD + and AMD/RPD- RPE samples

Differential gene expression analysis of single-cell RPE profiles using MAST (Additional file 2: Supplementary Data) identified 728 genes that differed significantly between AMD/RPD + and AMD/RPD- samples after multiple-testing correction (adjusted *p <* 0.05). Of these, 458 genes were upregulated and 270 downregulated in AMD/RPD + relative to AMD/RPD- (Fig. 2).

Compared to the AMD/RPD- cohort, the AMD/RPD + cohort showed upregulation of genes involved in extracellular matrice (ECM) remodelling and structural integrity, including collagen (e.g. *COL1A1*, *COL1A2*), *FN1*, *POSTN*, *HAPLN1*, *ITGBL1,* and *HSPG2*. Upregulation of *LINC01133* and *VIM* is consistent with features of epithelial-mesenchymal transition and cytoskeletal reorganisation. The canonical AMD-associated gene *HTRA1* was also higher in AMD/RPD +, consistent with its role in ECM regulation and AMD progression [62, 63]. Because variation at the *ARMS2/HTRA1* locus (rs11200638) is a major genetic risk factor for AMD, we compared genotype frequencies between groups: in the AMD/RPD- cohort, 45% of participants were heterozygous and 20% homozygous carriers, whereas in AMD/RPD + 45% were heterozygous and 42% homozygous carriers (*p* = 0.04518). Thus, although genotype differences at *ARMS2/HTRA1* could contribute to the observed transcriptional differences, they are unlikely to fully explain them, as none of the significantly differentially expressed genes are known direct targets of this locus. Additionally, increases in expression of mitochondrial (*MT-CYB*) and glycolytic genes (*LDHA*, *PGK1*, *FAM162A*), as well as *IGFBP2* were observed (Additional file 2: Supplementary Data).

Conversely, compared to AMD/RPD-, the addition of RPD was associated with relative downregulation of multiple mitochondrial transcripts, such as *MT-ATP8* (Complex V, associated with mitochondrial disorders, including retinitis pigmentosa [64]), and NADH-dehydrogenase subunits (*MT-ND2*, *MT-ND3*, *MT-ND4L*, *MT-ND5*, *MT-ND6*, which have been implicated in Leber Hereditary Optic Neuropathy [65]), alongside nuclear-encoded Complex I components (*NDUFA1*, *NDUFA3, NDUFA4, NDUFA13*, *NDUFB1*, *NDUFB2*, *NDUFS7*). Stress-response genes (*HSP90B1*, *HSPE1*, both of which participate in protein folding and endoplasmic reticulum stress responses and implicated in AMD pathogenesis [66, 67]), as well as the antioxidant enzyme *GPX4* and *PTGDS* (prostaglandin D₂ synthase) were also relatively reduced in AMD/RPD + compared with AMD/RPD- (Additional file 2: Supplementary Data).

**Fig. 3:**
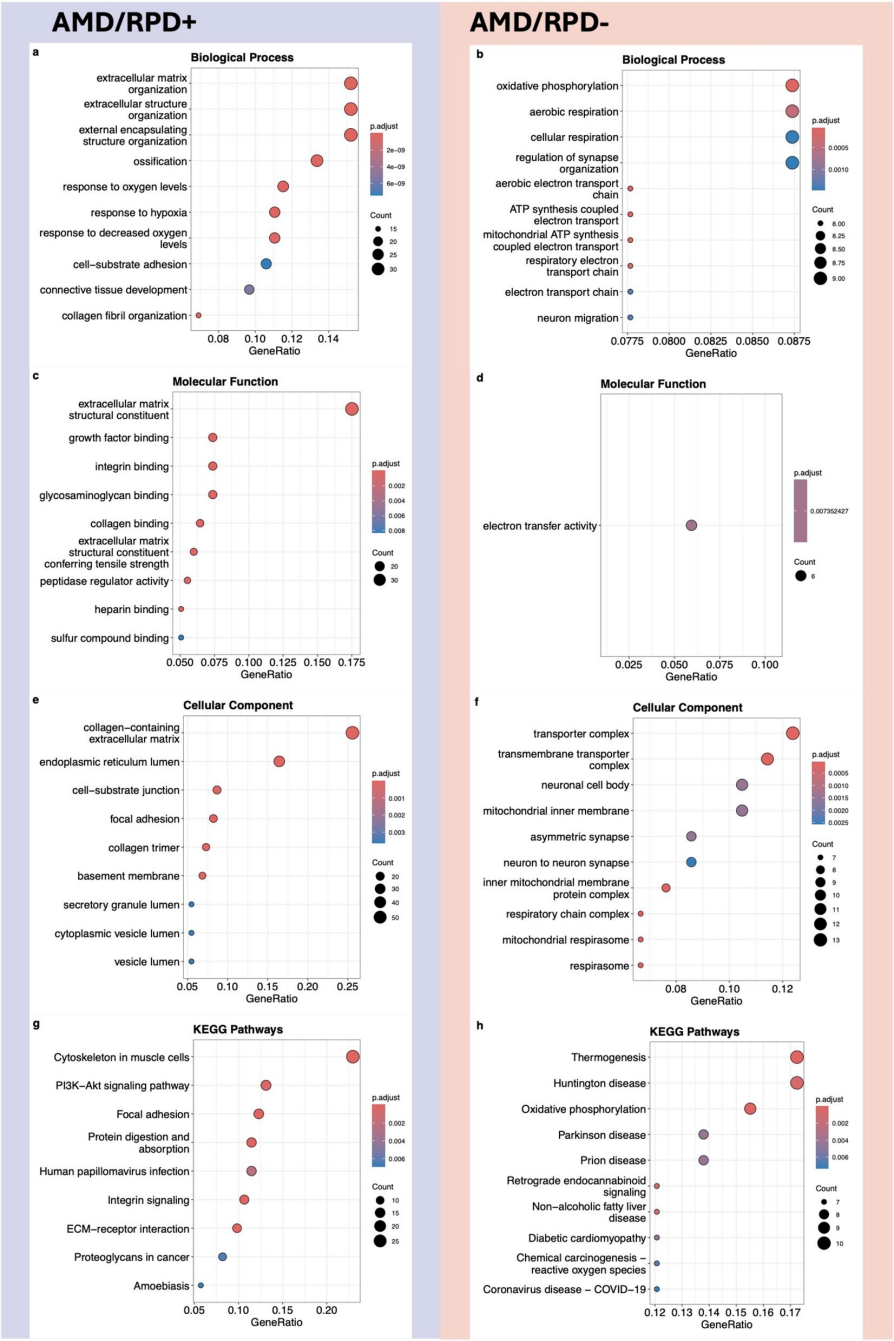
Enrichment analysis in each cohort. Pathway analysis showing the top 10 enriched Gene Ontology terms and KEGG pathway categories for genes upregulated in RPE cells from (**a**, **c**, **e**, **g**) AMD/RPD + compared to AMD/RPD-, and (**b**, **d**, **f**, **h**) AMD/RPD- compared to AMD/RPD + cohorts. Panels show the top enriched terms for (**a**, **b**) biological processes, (**c**, **d**) molecular functions, (**e**, **f**) cellular components, (**g**, **h**) and KEGG pathways categories. Pathways are ranked by gene ratio and adjusted *p*-value. Bubble size represents the number of genes associated with each term, and colour indicates the adjusted *p*-value (p.adjust). Pathways presented have ratios > 0.05

Overrepresentation analysis was used to assess pathway and disease associations of differentially expressed genes between the two cohorts [52], using the Gene Ontology [68] and Disease Ontology [69] databases (Fig. 3, Additional file 1: Fig. S3). In AMD/RPD +, enriched biological processes included ECM and structure organisation, cell-substrate adhesion, and responses to hypoxia and oxygen levels (Fig. 3a). Molecular function analysis showed enrichment for genes encoding proteins involved in ECM, integrin, collagen and growth factor binding, consistent with dynamic regulation of the extracellular environment and cellular attachment (Fig. 3c). Enriched cellular components included the collagen-containing ECM, focal adhesion, and cell-substrate junction (Fig. 3e). KEGG pathways further highlighted the overrepresentation of cytoskeletal, focal adhesion and ECM-receptor interaction pathways (Fig. 3g). Disease Ontology enrichment revealed genes upregulated were associated with bone conditions and cancer-related processes (Additional file 1: Fig. S3).

Compared to AMD/RPD- cells, pathways involved in mitochondrial energy metabolism, including oxidative phosphorylation, aerobic respiration, cellular respiration, and the electron transport chain, were underrepresented in AMD/RPD + (Fig. 3b). Consistently, genes associated with electron transfer activity were relatively reduced in AMD/RPD + cells (Fig. 3d). At the cellular component level, mitochondrial components such as the mitochondrial inner membrane, the respirasome and the respiratory chain complex were also underrepresented (Fig. 3f). These findings reflect relative enrichment of mitochondrial metabolic pathways in AMD/RPD- cells compared the AMD/RPD + cells, as further supported by KEGG pathways and Disease Ontology enrichment, which showed associations with mitochondrial-linked neurodegenerative diseases (e.g. Parkinson’s disease, Huntington’s disease, Fig. 3h, Additional file 1: Fig. S3).

Collectively, these data support two distinct transcriptional states within AMD. AMD/RPD + RPE cells show enrichment of ECM remodelling and hypoxia-responsive pathways, whereas mitochondrial and energy-metabolism processes, including oxidative phosphorylation, are relatively reduced compared with AMD/RPD-.

### Subpopulation-specific regulatory signatures in the RPE

To explore the impact of common genetic variation on gene regulation in the RPE, we conducted expression quantitative trait locus (eQTL) mapping within each subpopulation and identified 436 significant eQTLs (false discovery rate < 0.05). The number and distribution of eQTLs varied across cell subpopulations, with the highest burden observed in RPE3, RPE12, and RPE8 (Table 2, Additional file 1: Fig. S4, Additional file 2: Supplementary Data). We identified several eQTLs mapping to genes involved in diverse cellular processes, including signalling (*MAP2K2*), tRNA modification (*ELP5*), protein sorting (*RER1*), GPI-anchor remodeling (*PGAP2*), and lipid metabolism with links to oxidative stress (*TLCD5*). These eQTLs were not significantly associated with disease status. A subset of eQTLs showed significant interaction with disease status (SNP:disease *p <* 0.05), indicating regulatory effects that differed between AMD/RPD- and AMD/RPD + cohorts (Table 2, Additional file 1: Fig. S5). These include *AP3S1* (RPE3), *AIPL1* (RPE1), *FSTL5* (RPE8), *MSX2* (RPE3), *ZFPM2* (RPE4), *MC5R* (neuronal progenitors), and several long non-coding RNAs.

**Table 2:** Summary of eQTLs across RPE subpopulations

Population	Sig eQTLs*	Genes with sig eQTLs	Sig interactions with disease	Genes with Sig Interactions **
Overall RPE (early RPE, RPE)	36	*ELP5 (17), LINC00672 (17), AC126544.2 (17), KANSL1-AS1 (17), MAP2K2 (19)*	0	
Early RPE1	4	*PLD5 (1)*	0	
Early RPE2	13	*ABRA (8), PKP2 (12), AC132938.3 (17)*	0	
Neuronal Progenitors	14	*RNF17 (13), MC5R (18), PLVAP (19), AL118522.1 (20)*	5	*MC5R (18)*
RPE Progenitors	5	*C6orf201 (6), AC015540.1 (12), AL161757.5 (14)*	4	*C6orf201 (6),* *AL161757.5 (14)*
RPE1	14	*LINC02505 (4), DEC1 (9), AIPL1 (17), MAP2K2 (19)*	11	*LINC02505 (4), AIPL1* *(17)*
RPE2	49	*PEA15 (1), PURPL (5), AC008496.2 (5), AC006206.1 (12), AL159972.1 (13),* *AC092723.3 (16), MAP2K2 (19), AC005790.1 (19)*	7	*AC006206.1 (12),* *AC092723.3 (16)*
RPE3	118	*AP3S1 (5), MSX2 (5), CFTR (7), AL157394.3* *(10), RHOG (11), DHX33-DT(17), ELP5 (17), AC126544.2 (17), KIF19 (17)*	9	*MSX2 (5), AP3S1 (5), DHX33-DT(17)*
RPE4	3	*BZW2 (7), ZFPM2 (8)*	1	*ZFPM2 (8)*
RPE5	1	*ID2-AS1 (2)*	0	
RPE6	2	*LINC00571 (13), GPC6 (13)*	0	
RPE7	1	*MIR99AHG (21)*	0	
RPE8	58	*RER1 (1), FSTL5 (4), AC105213.1 (8), AL358790.1 (10), CPXM2 (10), MAP2K2 (19), EEF2* *(19), BRWD1-IT1 (21)*	16	*FSTL5 (4), AC105213.1* *(8)*
RPE9	25	*PGAP2 (11), AL159978.1 (13), AC091057.2 (15)*	2	*AL159978.1 (13)*
RPE10	20	*ZNF124 (1), LINC02150 (5), HMGN3-AS1 (6), USP45 (6), MAN1A1 (6), RHCG (15)*	9	*LINC02150 (5)*
RPE11	8	*AC112907.1 (3), TLCD5 (11), MYZAP (15), LINC00113 (21)*	1	*AC112907.1 (3)*
RPE12	101		0	

The number of significant eQTLs is shown for each subpopulation. Genes with significant eQTLs are listed with their chromosomal location. The number of loci showing significant genotype-disease interactions (*p <* 0.05) is reported separately, along with the corresponding genes

^*^Sig eQTL; *p* value < 0.05, false discovery rate < 0.05, homozygous alternate allele frequency ≥ 5

^**^Sig interaction; Genotype:disease interaction *p* value < 0.05

We next investigated whether previously reported SNPs influencing *ARMS2/HTRA1* expression, including variants recently associated with RPD (rs11200638, rs79641866/PARD3B, rs143184903/ITPR1, rs76377757/SLN, and the lncRNA gene *HTRA1-AS1*), were associated with altered gene expression in our dataset. rs11200638 was directly tested against both *HTRA1* and *ARMS2* but was not significant. The other reported SNPs were not present in our reference panel or were filtered during imputation quality control. However, nearby variants (rs849124 near *PARD3B*, rs4234561 near *ITPR1*, and rs4754244 near *SLN*) were tested, and none were significant. No variants were significantly associated with *HTRA1-AS1* expression. These results indicate that known AMD-associated and RPD-associated SNPs do not explain the elevated *HTRA1* transcript levels observed in the AMD/RPD + RPE cohort, and the corresponding increase in HTRA1 protein abundance described below. In summary, eQTLs capture both baseline regulatory variation and disease-specific effects, underscoring genetic contributions to differences in AMD subphenotypes.

### Differential protein expression between AMD/RPD + and AMD/RPD- RPE

**Fig. 4:**
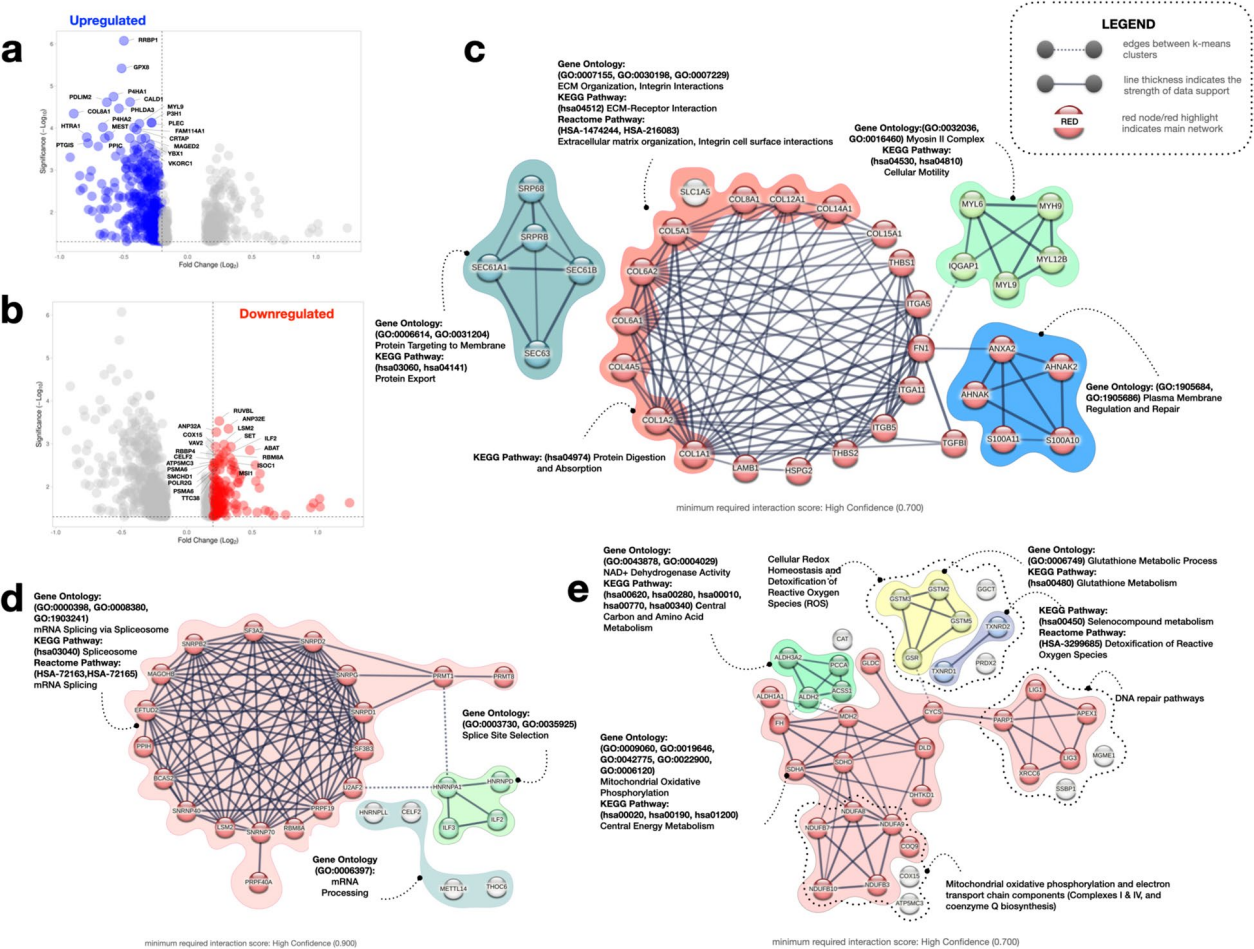
Differential protein expression between AMD/RPD + and AMD/RPD- RPE cells. **a**, **b** Volcano plots showing proteins significantly upregulated (a, blue) and downregulated (b, red) in AMD/RPD + relative to AMD/RPD- RPE cells (false discovery rate < 0.05, log₂ fold-change > 0.5). Selected proteins of interest are annotated. The distribution of abundances for the top differentially abundant proteins is shown in Fig. S6. **c**-**e** Protein–protein interaction networks of differentially abundant proteins identified by STRING (v12.0). **c** Upregulated proteins in AMD/RPD + form enriched clusters related to ECM organisation, cytoskeletal regulation, and membrane signalling. **d**-**e** Downregulated proteins in AMD/RPD + (upregulated in AMD/RPD-) cluster in modules related to mitochondrial metabolism, oxidative phosphorylation, RNA splicing, and antioxidant defence. **c**-**e** Edge thickness is proportional to STRING interaction confidence (minimum interaction score > 0.7). Functional clusters are colour-coded according to pathway enrichment (see Additional file 2)

To complement the transcriptomic analysis, we profiled the proteome of RPE cultures using tandem mass tag (TMT)-based mass spectrometry on bulk protein lysates. A total of 5,849 proteins were identified across six TMT runs, filtered at a 1% false discovery rate (Additional file 2: Supplementary data). Differentially expressed proteins were identified using standard statistical thresholds, and functional enrichment was performed using STRING v12.0 [70]. In total, 715 proteins were significantly differentially abundant between cohorts, with 502 upregulated and 213 downregulated in AMD/RPD + relative to AMD/RPD- (Fig. 4 a, b, Additional file 1: Fig. S6). Of these, 35 upregulated and 27 downregulated proteins have previously been associated with RPE dysfunction or AMD pathology. Canonical RPE markers were similarly expressed between groups (Additional file 1: Fig. S1). The top 16 differentially abundant proteins are shown in Additional file 1: Fig. S6 as violin plots to illustrate the distribution of TMT-normalised abundance values across biological replicates.

Many proteins were significantly upregulated in the AMD/RPD + cohort. These formed clusters involved in ECM organisation, cytoskeletal regulation, and membrane signalling (Fig. 4c). Notable ECM-associated proteins included COL1A1, COL1A2, COL4A5, COL6A2, FN1, and HSPG2, many of which are critical for ECM integrity and cell-ECM interactions. KEGG pathway analysis highlighted ECM-receptor interaction, protein digestion and absorption, and protein export, involving proteins such as SEC61A1, SEC61B, and SRP68. Increased expression extended to receptors for collagen and fibronectin, including ITGA11, associated with the blood-retinal barrier [71] and ITGB5, necessary for binding photoreceptor outer segments [72]. Components of the myosin motor complex (e.g. MYH9, MYL6, MYL9) were also increased, consistent with changes in cellular motility and structural tension. Among the 20 most significantly upregulated proteins in the AMD/RPD + group were several previously linked to AMD pathogenesis (VKORC1 [73], PLEC [74], COL8A1 [75, 76], HTRA1 [63]) or retinal disease (CALD1 [77], MYL9 [78], P3H1 [79], P4HA2 [80], RRBP1 [81]). Proteins involved in membrane integrity and calcium signalling, such as ANXA2, one of the most common proteins found in drusen and linked to inflammatory disease states [82, 83], as well as S100A10 and AHNAK, both associated with membrane repair, were also increased in abundance.

Several proteins were significantly reduced in AMD/RPD + relative to AMD/RPD- (Fig. 4a, Additional file 2: Supplementary data). Among those were proteins previously linked to glaucoma (ANP32A [84], VAV2 [85]) and retinal biology (MSI1 [86], RBM8A [87]). Consistent with this, functional enrichment analysis of proteins reduced in AMD/RPD + revealed overrepresentation of pathways related to RNA splicing and mRNA processing. These included multiple spliceosome-associated proteins (e.g. SNRNP70, RBM8A, PRPF19, U2AF2, SF3B3, HNRNPA1, CELF2) that were relatively less abundant in AMD/RPD + cells (Fig. 4d). Several of these have been implicated in retinal degenerative conditions (e.g. SNRPD1, SNRPD2, SNRPG, SNRPB2, SF3A2) [72, 73]. Pathways linked to DNA repair and cellular maintenance were also less represented in the AMD/RPD + cohort, including proteins involved in base excision repair (e.g. PARP1, LIG1, LIG3, APEX1) and antioxidant defence (e.g. PRDX2, GSR, CAT) (Fig. 4e). Multiple aldehyde dehydrogenases (e.g. ALDH2, ALDH3A2, ALDH1A1) showed lower abundance, consistent with reduced capacity to detoxify reactive aldehydes and to mitigate lipid peroxidation (Fig. 4e). Similarly, mitochondrial metabolism pathways were downregulated in AMD/RPD +, with decreased abundance of TCA cycle enzymes (DLD, MDH2, FH) and glutathione metabolism proteins (GSTM2, GSTM3, GSTM5, GGCT), consistent with diminished antioxidant and bioenergetic capacity compared to the AMD/RPD- cells (Fig. 4e). Consistent with the transcriptomic findings, proteins involved in mitochondrial respiration and oxidative phosphorylation were also reduced in AMD/RPD + (Fig. 4e). These include Complex I subunits (NDUFA8, NDUFA9, NDUFB3, NDUFB10), cytochrome oxidase biosynthesis (COX15), and electron transport components (COQ9, ATP5MC3). Several of these proteins have previously been implicated in the pathophysiology of geographic atrophy in RPE cells [40].

### Protein-level regulatory signatures in the RPE

**Fig. 5:**
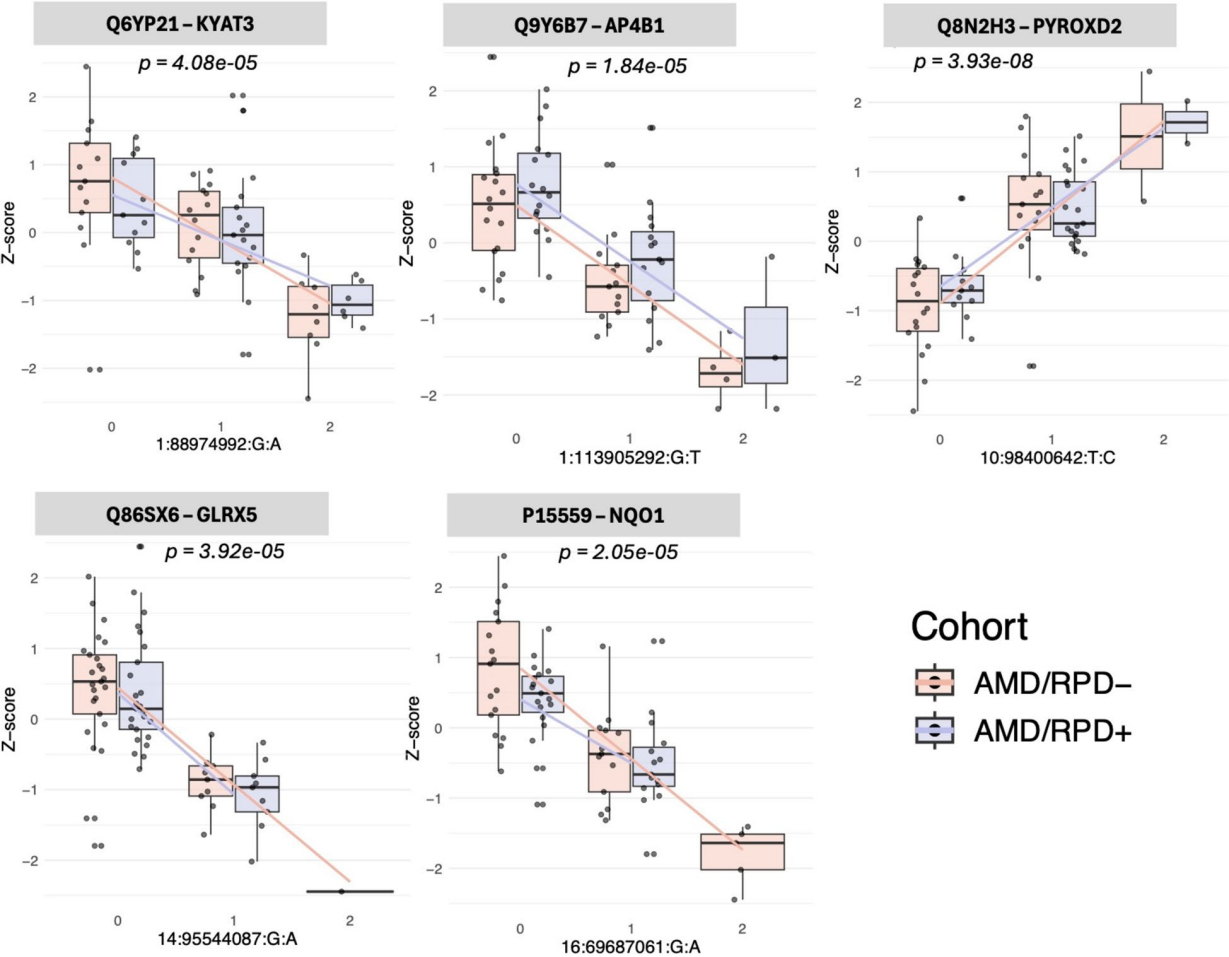
Protein quantitative trait loci in RPE cells. Representative pQTLs showing association between genotype and protein abundance for KYAT3 (Q6YP21), AP4B1 (Q9Y6B7), PYROXD2 (Q8N2H3), GLRX5 (Q86SX6), and NQO1 (P15559). Boxplots display rank-normalized protein abundance (y-axis) stratified by genotype (x-axis), with data shown separately for AMD/RPD- (red) and AMD/RPD + (blue) cohorts. Linear regression fits are overlaid to illustrate allele dosage effects

To explore the genetic regulation of protein abundance, we performed protein quantitative trait locus (pQTL) analyses. We identified five significant pQTLs present within both AMD/RPD- and AMD/RPD + cohorts (false discovery rate < 0.05; Tables 3, Additional file 1: Table S1, Fig. 5). No differences were observed between AMD/RPD- and AMD/RPD + cohorts. Four pQTLs mapped to proteins with mitochondrial functions, including KYAT3 (tryptophan metabolism), PYROXD2 (oxidoreductase), GLRX5 (iron-sulfur cluster biogenesis), and NQO1 (redox cycling). The only non-mitochondrial hit was AP4B1, an adaptor protein complex subunit involved in intracellular trafficking.

**Table 3:** Significant pQTLs

Gene	Protein (Uniprot ID)	SNP (rsID)	Slope (β)	Adjusted β *p*-value	False discovery rate
*KYAT3*	Q6YP21	rs35594043	−0.784461	4.07702e-05	0.0435
*AP4B1*	Q9Y6B7	rs971173	−0.836572	1.84237e-05	0.0365
*PYROXD2*	Q8N2H3	rs6584194	1.14271	3.92864e-08	0.0002
*GLRX5*	Q86SX6	rs11628901	−1.16739	3.92053e-05	0.0435
*NQO1*	P15559	rs3169315	−0.876558	2.05268e-05	0.0365

Gene symbol, UniProt identifier of the encoded protein, SNP ID, effect size (β), adjusted β *p*-value, and false discovery rate for each association

### Integrating genetic regulation with disease risk

**Fig. 6:**
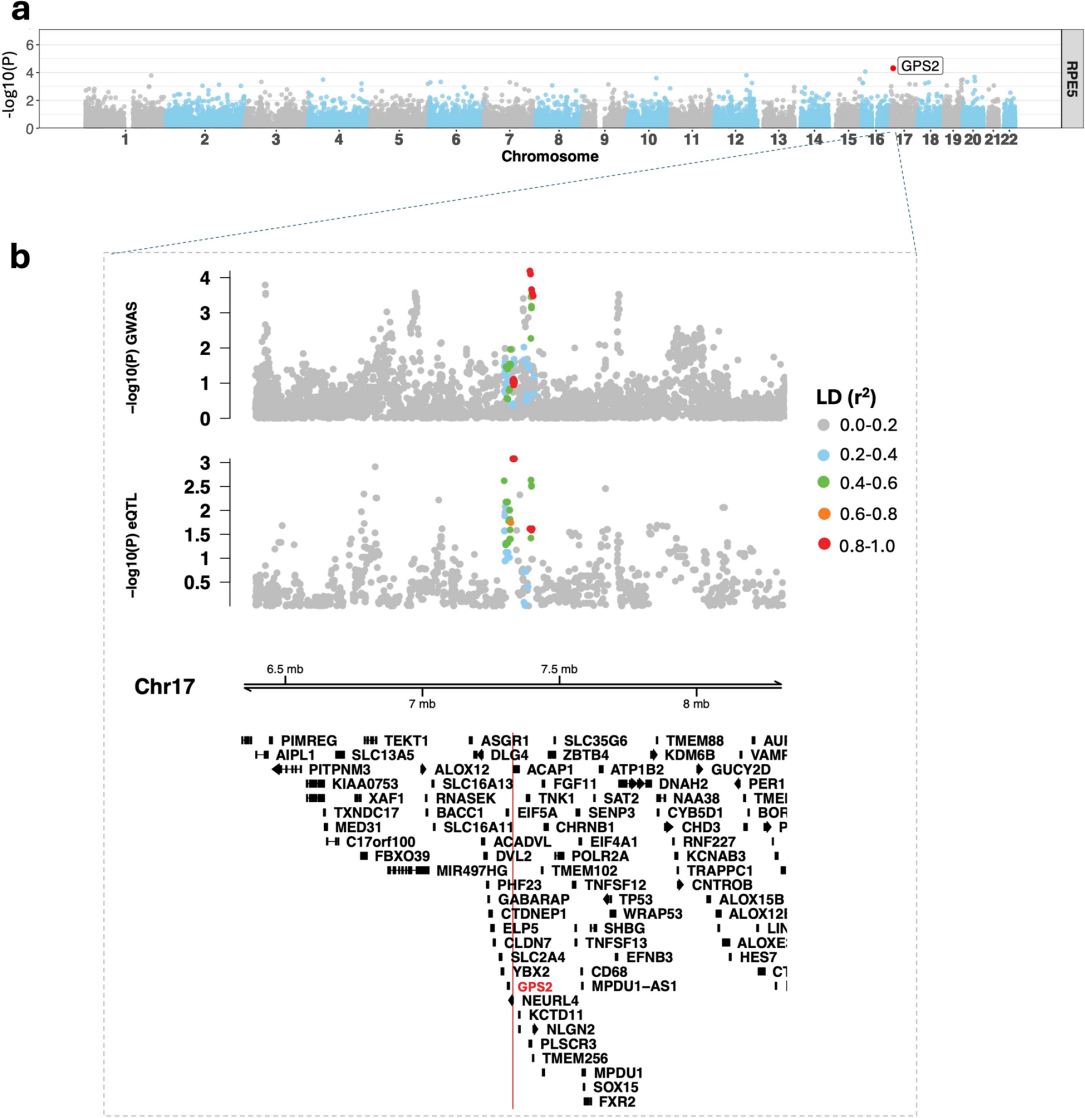
Colocalisation at the *GPS2* TWAS signal. **a** Gene-level manhattan plot of TWAS results showing *GPS2* signal in RPE5 population (TWAS *P* value 4.89e-05). **b** Locus zoom plots showing colocalisation of GWAS and eQTL signatures for the *GPS2* region (± 1 MB window) on chromosome 17. The upper panel shows GWAS association (-log10(P)) by genomic position (lead variant rs3744549). The middle panel shows eQTL association (-log10(P)) across the same region (lead variant rs3809816). Points are coloured by linkage disequilibrium (r^2^) with the lead GWAS variant. The lower panel shows gene annotations for the region, with *GPS2* indicated

To further integrate genetic and transcriptomic signals, a TWAS was performed as a complementary approach to eQTL and pQTL analyses, linking genetic variation to disease through predicted gene expression and thereby prioritising candidate genes beyond individual QTL effects. Sixty gene-trait associations reached significance across the overall RPE population and subpopulations (Fig. 6, Additional file 1: Fig. S7, Additional file 2: Supplementary data). Among the significant associations, several genes encode proteins involved in mitochondrial metabolism and homeostasis, including *GPS2* (Fig. 6), *LYRM4*, *ALDH9A1* and *EGLN3/PHD3* (Additional file 1: Fig. S7). Additional significant loci included *SMAD3* and *TCF21* (Additional file 1: Fig. S7), genes linked to the ECM. Other associated genes with reported retinal expression or function included *DGCR8 * [88, 89], *FEZF1 * [90], *PSMC1 * [91], and *TENM4* [92].

### Functional analyses

Both transcriptomic and proteomic analyses revealed differences in ECM organisation and cell-substrate adhesion pathways between AMD cohorts with and without RPD. To test whether these molecular signatures corresponded to functional differences, we performed assays in iPSC-derived RPE cells generated from control donors (selected from our previous studies [40]), and from AMD/RPD- and AMD/RPD + donors. Transmission electron microscopy did not show consistent ultrastructural differences between the three groups (Additional file 1: Fig. S8). No statistically significant differences in baseline TEER were observed between cohorts (Additional file 1: Fig. S9), indicating comparable barrier integrity under maintenance conditions.

**Fig. 7:**
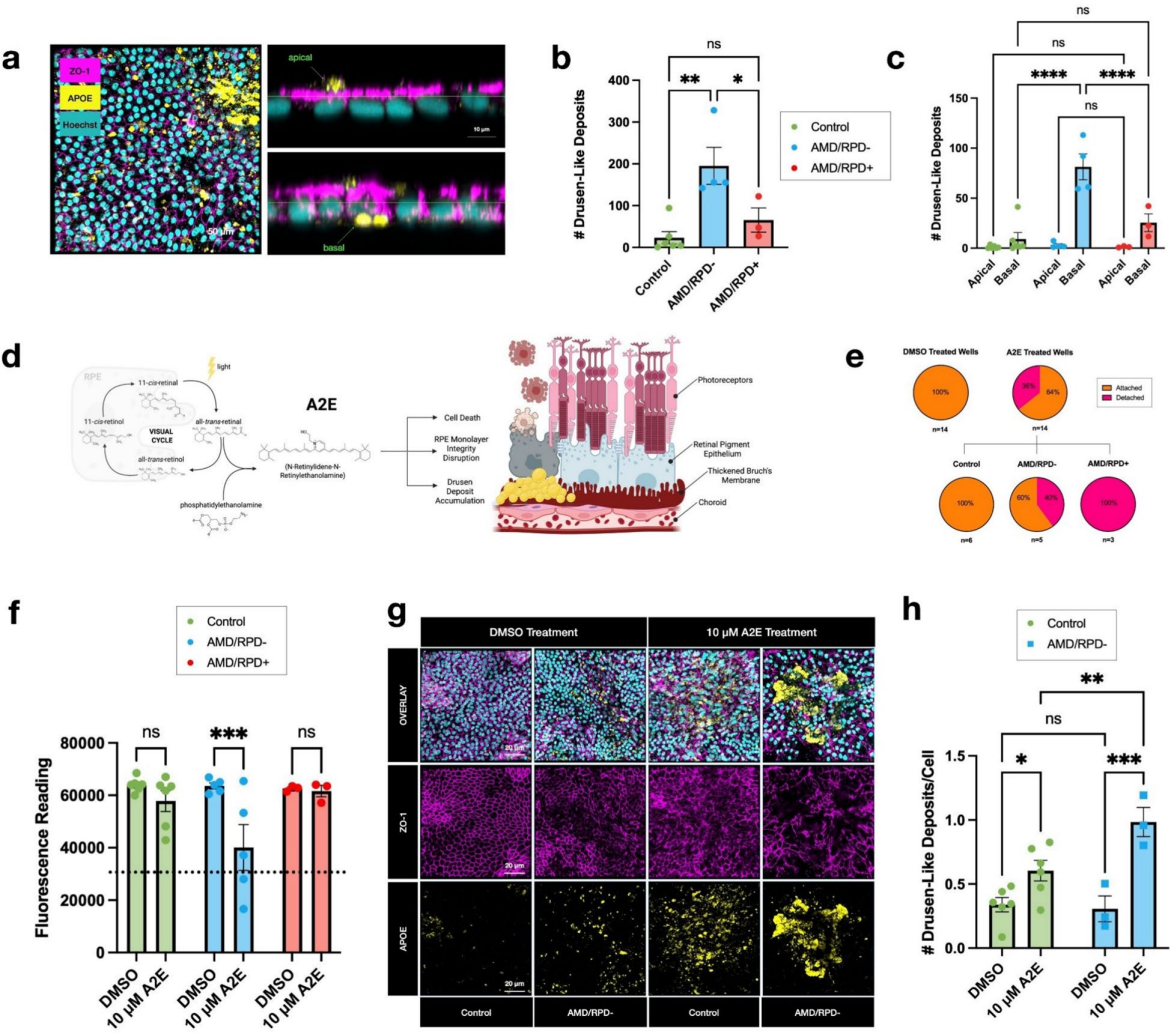
Functional characterisation of iPSC-derived RPE cells. **a** Left: Representative high-resolution Z-stack image of iPSC-derived RPE cells stained for ZO-1, APOE and Hoechst. This panel was previously published in [45] and is reproduced here for illustrative purposes under CC BY-NC-ND licence. Scale bar: 50 µm. Right: orthogonal view of Z-stack, lime horizontal bar to show apical versus basal distinction. Scale bar: 10 µm. **b** Number of drusen-like deposits in control and AMD lines. Data are mean ± SEM of triplicate values per well from all lines. Control: 9.1 ± 10.2, *n* = 6 lines; AMD/RPD-: 195.2 ± 88.8, *n* = 4 lines; AMD/RPD + : 65.4 ± 50.3, *n* = 3 lines; *p*-values: AMD/RPD- versus AMD/RPD + : 0.0408, AMD/RPD- versus Control: 0.0030, AMD/RPD + versus Control: 0.5897. **c** Number of apical versus basal drusen-like deposits. Mean deposit number < 50 µm.^3^ ± SEM: AMD/RPD-: apical: 3.0 ± 2.9, basal: 81.3 ± 25.9, *n* = 4 lines; AMD/RPD + : apical: 1.2 ± 0.7, basal:25.4 ± 15.4, *n* = 3 lines; Control: apical: 1.8 ± 1.6, basal: 2.7 ± 2.9 *n* = 6 lines; *p*-values for basal drusen-like deposit comparison: AMD/RPD- vs. AMD/RPD + : < 0.0001, AMD/RPD- vs. Control: < 0.0001; AMD/RPD + vs Control: 0.2390. **d** Schematic of the impact of A2E on RPE cells; build-up of A2E in the cells induces cell death, RPE monolayer integrity disruption, and drusen deposit accumulation. Created in BioRender. Hall, J. (2026) under CC BY 4.0 license agreement #: SW28ZE3T50 https://BioRender.com/cln2ln4. **e** Pie chart breaking down detachment in treated wells. **f** PrestoBlue viability readings. Mean fluorescence value ± SEM: AMD/RPD-: DMSO: 63,592.0 ± 2560, A2E: 40,113.8 ± 19,513.8, *n* = 5 lines; AMD/RPD + : DMSO: 62,574.3 ± 1067.9, A2E: 61,580.0 ± 3810.4, *n* = 3; lines Control: DMSO: 63,950.0 ± 2784.1, A2E: 57,841.5 ± 9841.6, *n* = 6 lines; *p*-values for DMSO versus A2E treatment: AMD/RPD-: < 0.0010, AMD/RPD + : 0.9020, Control: 0.2910. Dotted line marks viability cutoff; samples below this line were excluded in drusen-like deposit analysis in (h). **g** DMSO- and A2E-treated RPE exhibiting tight junction marker ZO-1 (pink), drusen deposit marker APOE (yellow) and nuclear stain Hoechst. Scale bar: 20 µm. **h** Number of drusen-like deposits normalized to cell count. Mean normalized deposit number ± SEM: AMD/RPD-: DMSO: 0.3 ± 0.2, A2E: 1.0 ± 0.2, *n* = 3; Control: DMSO:0.3 ± 0.1, A2E: 0.6 ± 0.3 *n* = 6 lines; *p*-values for drusen-like deposit comparison: Control: DMSO vs. A2E: 0.0337; AMD/RPD-: DMSO versus A2E: 0.0008; Control vs. AMD/RPD-: DMSO: 0.9449, A2E: 0.0080. **b**, **c**, **f**, **h** Statistical significance was established by 2-way-ANOVA followed by Tukey’s Multiple Comparisons Test, **** *p <* 0.0001; *** *p <* 0.001; ** *p <* 0.01; * *p <* 0.05

As we previously reported [45], all three iPSC-derived RPE cohorts formed drusen-like deposits in vitro. Among the AMD-derived cells, the AMD/RPD- lines produced more basal deposits than either the control or AMD/RPD + groups (Fig. 7a-c, Additional file 3: additional video). To assess stress susceptibility, RPE cells were exposed to the toxic N-Retinylidene-N-Retinylethanolamine (A2E, 10 µM, 7 days), which accumulates with aging and AMD, and induces oxidative and lysosomal stress (Fig. 7d). After treatment, AMD-derived RPE wells showed widespread detachment (absent in controls), most markedly in the AMD/RPD + group, consistent with compromised monolayer integrity (Fig. 7e). Despite this monolayer disruption, overt cell death was observed only in A2E-treated AMD/RPD- cultures, whereas AMD/RPD + cells largely remained viable (Fig. 7f). Because extensive monolayer loss in AMD/RPD + wells precluded reliable quantitative analysis of intact areas, drusen-like deposits could not be measured in this cohort following A2E exposure. However, A2E exposure significantly increased drusen-like deposits in the remaining attached AMD/RPD- cultures compared with controls (Fig. 7g, h). Collectively, these findings indicate that the two AMD subphenotypes respond differently to stress in vitro: AMD/RPD + cells are more susceptible to monolayer disruption under stress, whereas AMD/RPD- cells exhibit greater cell loss. These differences are consistent with their respective molecular profiles, with ECM-associated pathways more prominent and mitochondrial/oxidative stress pathways underrepresented in AMD/RPD + relative to AMD/RPD- cells.

## Discussion

This study delineates molecular and functional differences between two clinically defined subphenotypes of AMD: with and without RPD. All differential expression and QTL analyses included age and sex as covariates to mitigate confounding by these factors. AMD/RPD + RPE cells were characterised by enrichment of ECM remodelling, adhesion and hypoxia-responsive pathways, together with relative depletion of mitochondrial pathways compared to AMD/RPD- RPE cells. Although both subphenotypes exhibited molecular signatures consistent with mitochondrial dysfunction, the relative balance of ECM-associated versus mitochondrial processes differed between them.

Despite sharing features such as drusen, AMD patients with RPD are clinically recognised to have worse visual function and a greater risk of progression to late-stage AMD than patients without RPD [10, 14]. RPD has been associated with alterations in macular structure and retinal integrity, which have been linked to functional impairment in several studies [10]. Although our iPSC-derived RPE model does not recapitulate this tissue context, AMD/RPD + cells display enrichment of hypoxia-responsive and ECM-related pathways under shared in vitro conditions. This suggests that the observed differences are unlikely to depend solely on the local retinal microenvironment and could reflect intrinsic donor-specific regulatory architecture. If present in vivo, such pathway differences could influence neurosensory retina-RPE interactions and thereby contribute to tissue alterations observed in RPD.

Integration of genetic data provides additional context for these findings. Most eQTLs were not significantly associated with disease status, and given that all donors had AMD, they are unlikely to represent primary risk factors for disease onset. Instead, they may reflect regulatory variation contributing to phenotypic heterogeneity within AMD.

A subset of eQTLs showed significant disease-interactions, indicating that the direction or magnitude of regulatory effects differs between the AMD/RPD- and AMD/RPD + cohorts. Several map to genes with established relevance to retinal cell biology, including *AP3S1*, which is involved in intracellular trafficking and Golgi-to-lysosome cargo sorting [93], and melanocyte trafficking and melanogenesis [94]. This is pertinent to the RPE, where trafficking pathways support melanosome biology and lysosomal processing of photoreceptor outer segments, processes linked to drusen-associated pathology [95]. Additional disease-interacting loci include *AIPL1*, previously implicated in inherited retinopathies [96]; *FSTL5*, linked to BMP signalling and ECM regulation; and the developmental transcription factors *MSX2* and *ZFPM2*. Signals involving developmental regulators could reflect altered transcriptional states in AMD RPE cells, although contributions from residual immaturity in iPSC-derived cells cannot be excluded.

In the pQTL analysis, significant associations mapped to proteins with established roles in mitochondrial redox balance and iron-sulfur cluster biology. These include KYAT3 (implicated in retinal neuroprotection [97, 98]), PYROXD2 (associated with geographic atrophy in RPE cells [40]), GLRX5 and NQO1 (implicated in AMD [99]). AP4B1 functions in endo-lysosomal trafficking pathways that influence cellular iron handling. Considered alongside GLRX5, which regulates mitochondrial iron-sulfur cluster biogenesis, these findings suggest complementary components of iron homeostasis. Importantly, these pQTL effects were shared across AMD/RPD- and AMD/RPD + cohorts, consistent with a common genetic modulation of mitochondrial and iron-handling processes within AMD, rather than with subphenotype-specific protein-level effects. The TWAS analysis extended these observations by linking genetically predicted gene expression to RPD risk. In addition to mitochondrial and stress-response loci (e.g. *GPS2*, *LYRM4*, *ALDH9A1*, *EGLN3/PHD3*), TWAS identified associations at *SMAD3* and *TCF21*, transcription factors within TGF-β regulatory networks [100].

At the transcriptomic and proteomic levels, the two subphenotypes diverged: AMD/RPD + RPE cells were characterised by enrichment of ECM remodelling and adhesion-related pathways, and a relative underrepresentation of mitochondrial and oxidative phosphorylation processes, compared to AMD/RPD- cells.

Collectively, these results suggest two genetic themes: shared modulation of mitochondrial and iron-handling processes across AMD cohorts, consistent with a shared axis of metabolic vulnerability in AMD [101, 102]; and additional ECM-associated loci emerging specifically in AMD/RPD +. The transcriptomic and proteomic data are consistent with this pattern, showing greater engagement of ECM and adhesion pathways in AMD/RPD + cells.

Because the present comparison is restricted to AMD cohorts, absolute differences relative to healthy RPE cannot be inferred. However, these data suggest that the distinction between AMD/RPD + and AMD/RPD- relates more to differences in downstream pathways engaged in each subphenotype than to broad differences in underlying genetic architecture. Consistent with this interpretation, our previous work on RPE cells in geographic atrophy demonstrated mitochondrial impairment relative to healthy controls [40], supporting the view that metabolic dysregulation is a common component of AMD pathobiology across clinical presentations, although its relative prominence may differ across subphenotypes.

## Conclusions

Taken together, these findings support a layered model in which shared metabolic vulnerability coexists with differential emphasis on downstream pathways across subphenotypes. Within this framework, AMD/RPD + shows greater engagement of ECM, cytoskeletal, and adhesion pathways, as well as increased susceptibility to stress-induced monolayer disruption under the conditions tested. These trajectories are not mutually exclusive, and mitochondrial dysfunction likely contributes to both phenotypes. This interpretation aligns with existing models of AMD pathogenesis that emphasise RPE dysfunction as a primary driver in conventional AMD [103].

These differences have practical implications. First, our findings challenge the view that the presence of RPD represents a cumulative extension of conventional AMD pathology. Second, they highlight the limitations of AMD classifications based solely on conventional drusen. Integrating molecular profiling into AMD subclassification may improve prognostic resolution and therapeutic targeting.

Our findings also underscore the strengths and limitations of iPSC-derived RPE modelling. While these cultures effectively capture cell-intrinsic responses, they do not reflect the influence of surrounding retinal, vascular, or immune components that are likely relevant to RPD formation. Furthermore, quantitative assessment of junctional organisation and adhesion dynamics will be required to define the mechanisms underlying monolayer disruption in AMD/RPD + RPE cells. Finally, while mitochondrial signatures were evident across subphenotypes, the functional assays presented here primarily address epithelial stability under stress. Complementary mitochondrial functional analyses will be valuable in future work to further define their contribution to AMD subphenotypes.

## Supplementary Information

Additional file 1. Additional figures and table.

Additional file 2. All datasets.

Additional file 3. Additional video.

## Data Availability

All data supporting the findings of this study are available within the paper and its additional files. All sequencing and processed transcriptomic data generated in this study have been deposited in the ArrayExpress database under accession identifier E-MTAB-16679 (https://www.ebi.ac.uk/arrayexpress) [104], and the mass spectrometry raw file and search results have been deposited in PRIDE via the ProteomeXchange Consortium [105] (https://www.ebi.ac.uk/pride) under accession PXD070797 (http://www.ebi.ac.uk/pride) [106]. All codes used in this study are publicly available at GitHub ( https://github.com/powellgenomicslab/AMD_RPD/tree/main) [107]. Requests for materials and correspondence should be addressed to Alice Pébay.

## Abbreviations

AMD: Age-related macular degeneration
AMD/RPD-: AMD with only conventional drusen
AMD/RPD +: AMD with conventional drusen and with extensive reticular pseudodrusen
CNV: Copy number variant
CPM: Count per million
ECM: Extracellular matrix
eQTL: Expression quantitative trait locus
FC: Fold change
GWAS: Genome-wide association study
iPSCs: Induced pluripotent stem cells
A2E: N-Retinylidene-N-Retinylethanolamine
POS: Photoreceptor outer segments
pQTL: Protein quantitative trait locus
PSMs: Peptide-spectrum matches
QTL: Quantitative trait locus
RA: Relative abundance
RPD: Reticular pseudodrusen
RPE: Retinal pigment epithelium
sig: Significant
TWAS: Transcriptome-wide association study
TMT: Tandem mass tag
UMAP: Uniform manifold approximation and projection
UMI: Unique molecular identifier
