# Correction: Patient induced pluripotent stem cells identify specificities of a reticular pseudodrusen phenotype in age-related macular degeneration

**DOI:** 10.1186/s13073-026-01728-5

**Published:** 2026-07-22

**Authors:** Jenna C. Hall, Kavitha Krishna Sudhakar, Maciej Daniszewski, Anne Senabouth, Carla J. Abbott, Helena H. Liang, Himeesh Kumar, Grace E. Lidgerwood, Mehdi Mirzaei, Jessica YW Ma, Trevor Atkeson, Yumiko Hirokawa, Emeline F. Nandrot, Alexander Barnett, Chantal Cazevieille, Gaël Manes, Simon Mountford, Philip Thompson, Erica L. Fletcher, Zhichao Wu, Melanie Bahlo, Brendan R. E. Ansell, Daniel Paull, Alex W. Hewitt, Robyn H. Guymer, Joseph E. Powell, Alice Pébay

**Affiliations:** 1 Department of Anatomy and Physiology, The University of Melbourne, Parkville, VIC 3010 Australia; 2 Translational Genomics, Garvan Institute of Medical Research, Sydney, NSW 2010 Australia; 3 Centre for Eye Research Australia, Royal Victorian Eye and Ear Hospital, East Melbourne, VIC 3002 Australia; 4 Department of Surgery, Ophthalmology, The University of Melbourne, East Melbourne, VIC 3002 Australia; 5 Faculty of Medicine, Health and Human Sciences, Macquarie Medical School, Macquarie University, Sydney, NSW 2109 Australia; 6 Sorbonne Université, CNRS, INSERM, Institut de La Vision, Paris, 75012 France; 7 Menzies Institute for Medical Research, University of Tasmania, Hobart, TAS 7000 Australia; 8 Université de Montpellier, INSERM U1298, Institut Des Neurosciences de Montpellier, Hôpital Saint-Eloi, Montpellier, France; 9 Monash Institute of Pharmaceutical Sciences, Monash University, Parkville, VIC 3058 Australia; 10 Population Health and Immunity Division, Walter and Eliza Hall Institute of Medical Research, Parkville, VIC 3010 Australia; 11 Department of Medical Biology, The University of Melbourne, Parkville, VIC 3010 Australia; 12 The New York Stem Cell Foundation Research Institute, New York, USA; 13 School of Medicine, University of Tasmania, Hobart, TAS 7005 Australia; 14 CellTellus Laboratory, Melbourne, VIC 3000 Australia; 15 UNSW Cellular Genomics Futures Institute, University of New South Wales, Sydney, NSW 2052 Australia; 16 Department of Surgery, Royal Melbourne Hospital, The University of Melbourne, Parkville, VIC 3010 Australia

**Correction: Genome Med 18, 54 (2026)**

**https://doi.org/10.1186/s13073-026-01658-2**

The original publication of this article was missing an disclaimer in Fig. 7a that the panel was re-used (with permissions). The incorrect and correct information is shown in this correction article, the original article has been updated.

Incorrect panel 7a wording

a Left: representative high-resolution Z-stack ZO-1, APOE, Hoechst. Scale bar: 50 μm. Right: orthogonal view of Z-stack, lime horizontal bar to show apical versus basal distinction. Scale bar: 10 μm.

Correct panel 7a wording

a Left: Representative high-resolution Z-stack image of iPSC-derived RPE cells stained for ZO-1, APOE and Hoechst. This panel was previously published in [45] and is reproduced here for illustrative purposes under CC BY-NC-ND licence. Scale bar: 50 μm. Right: orthogonal view of Z-stack, lime horizontal bar to show apical versus basal distinction. Scale bar: 10 μm.

