# Supplementary material for: Patient induced pluripotent stem cells identify specificities of a reticular pseudodrusen phenotype in age-related macular degeneration
Source: Genome Med. 2026 May 10;18:54. doi: 10.1186/s13073-026-01658-2 (PMC13157670; doi:10.1186/s13073-026-01658-2)
Supplement: Supplementary file 1 — Additional file 1. Additional figures and table. [file 13073_2026_1658_MOESM1_ESM.docx]

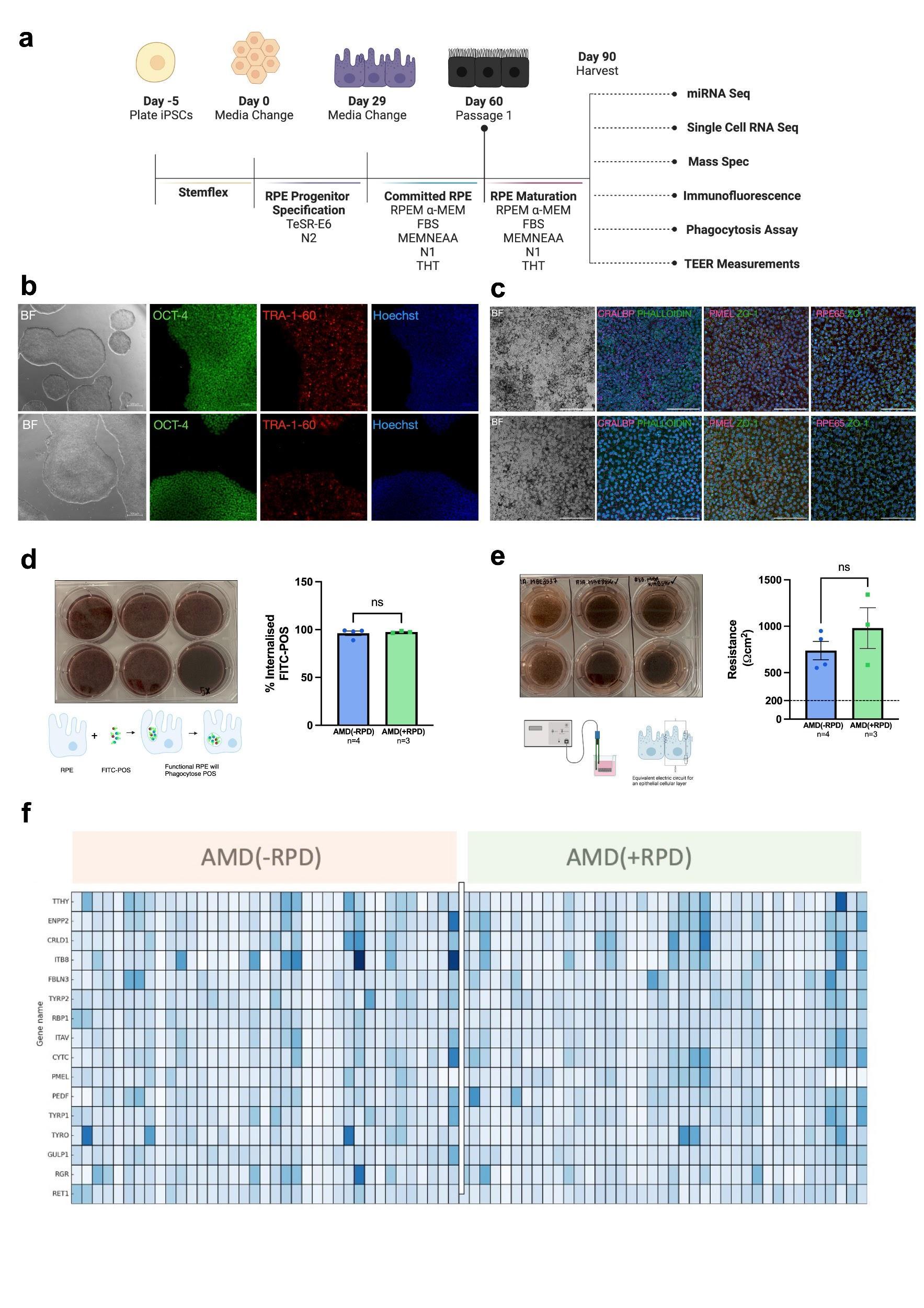


**Fig S1.** **Quality control.**

(**a**) Schematic overview of the experimental workflow (created with [BioRender.com](http://biorender.com)). (**b**) Quality control of generated iPSC lines showing representative brightfield (BF) and immunofluorescence images for OCT-4, TRA-1-60, and Hoechst 33342 in a AMD/RPD- line (MBE-03556, top) and an AMD/RPD+ line (MBE-01986, bottom). Scale bars: 500 µm (BF), 100 µm (fluorescence). Images are representative of all lines. (**c**) Characterisation of iPSC-derived RPE cells from the same two lines, showing BF and immunostaining for phalloidin (magenta) and CRALBP (green); PMEL (magenta) and ZO-1 (green); RPE65 (magenta) and ZO-1 (green). Scale bars: 100 µm. (**d**) Phagocytosis assay. Representative pigmented RPE cells and schematic (BioRender) showing uptake of FITC-labelled photoreceptor outer segments (POS). Functional assay results for AMD/RPD- (MBE-03556, MBE-03556, MBE-03556, MBE-03556) and AMD/RPD+ (TOB-01986, TOB-01986, TOB-01986) lines, presented as mean % engulfment ± SEM from technical triplicates of each unique iPSC-derived RPE line. Significance was assessed by t-test (p < 0.05). (**e**) Transepithelial electrical resistance (TEER) assay. Representative pigmented RPE cells on transwell inserts and schematic (BioRender) of the TEER setup. Resistance values are shown for AMD/RPD- (MBE-03556, MBE-03556, MBE-03556, MBE-03556) and AMD/RPD+ (TOB-01986, TOB-01986, TOB-01986) lines. Each dot represents the average of technical triplicates for each iPSC-derived line. Data are shown as mean ± SEM, with statistical significance determined by *t*-test (p < 0.05). (**f**) Heatmap of RPE canonical marker expression from mass spectrometry across all AMD/RPD- and AMD/RPD+ lines. Each rectangle represents one line; colour intensity reflects raw abundance values from minimal (light blue) to maximal (dark blue).


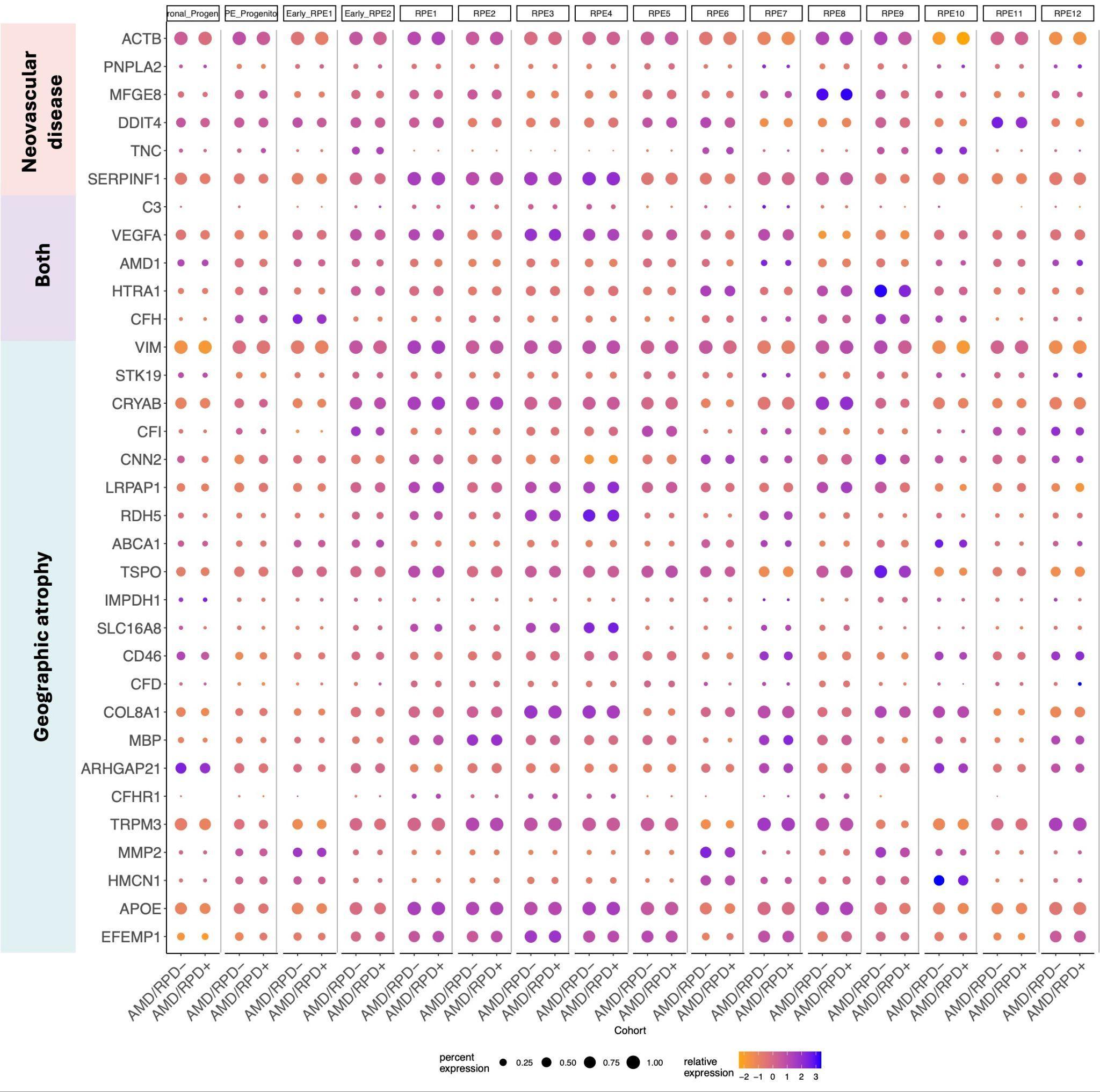


**Fig S2.** **Genes associated with cell subpopulations and their expression in AMD/RPD- and AMD/RPD+ cells.** Dotplot representation of single-cell expression profiles for genes linked to AMD. Plots show scaled average expression (z-scores; color scale) and the percentage of cells within each cluster expressing the gene (dot size).


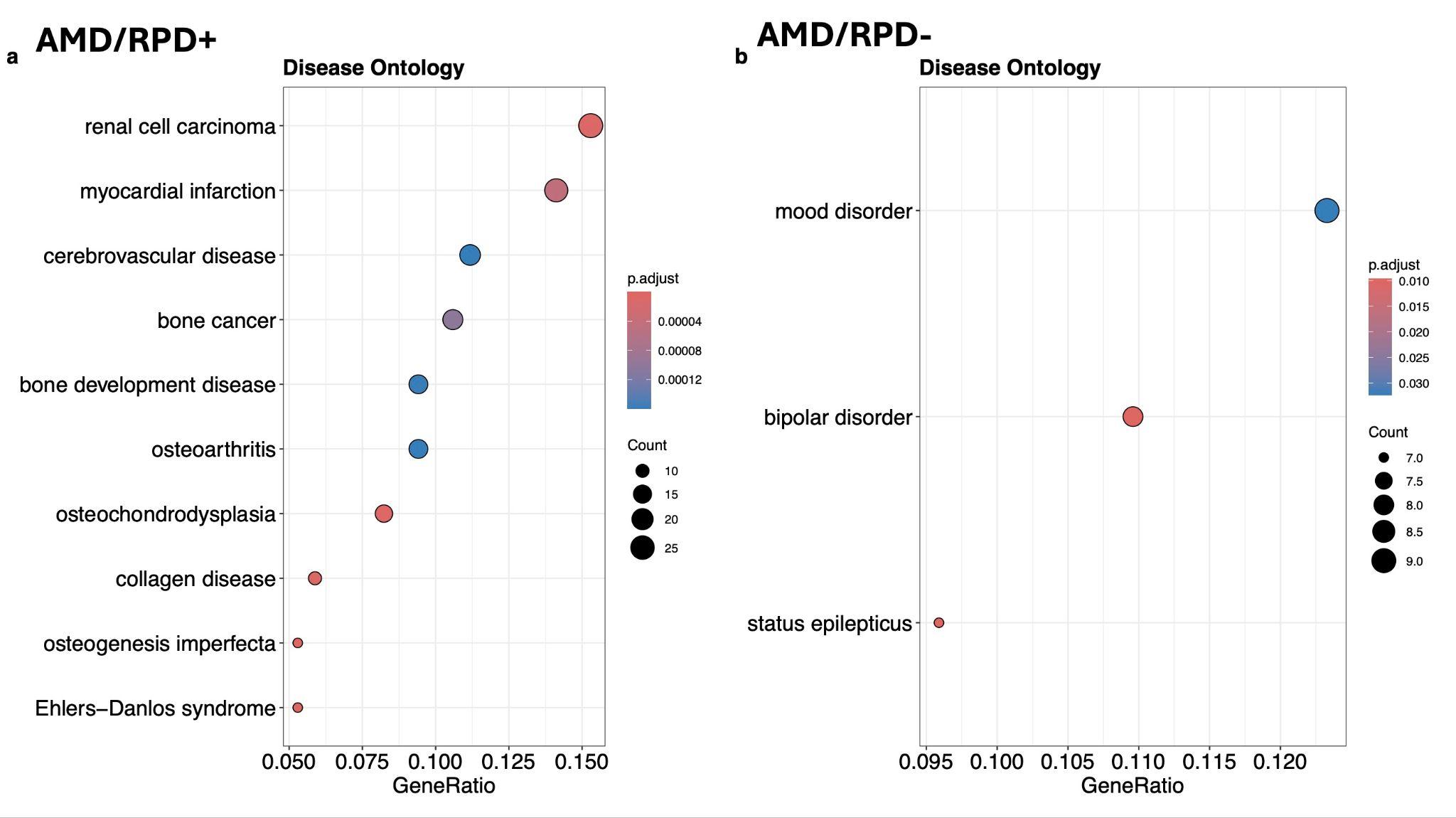


**Fig S3. Disease Ontology enrichment analysis of differentially expressed genes in AMD/RPD+ and AMD/RPD- RPE cells.** The top 10 enriched Disease Ontology categories are shown. The number of genes associated with each term, and colour indicates the adjusted p value (p.adjust).

**
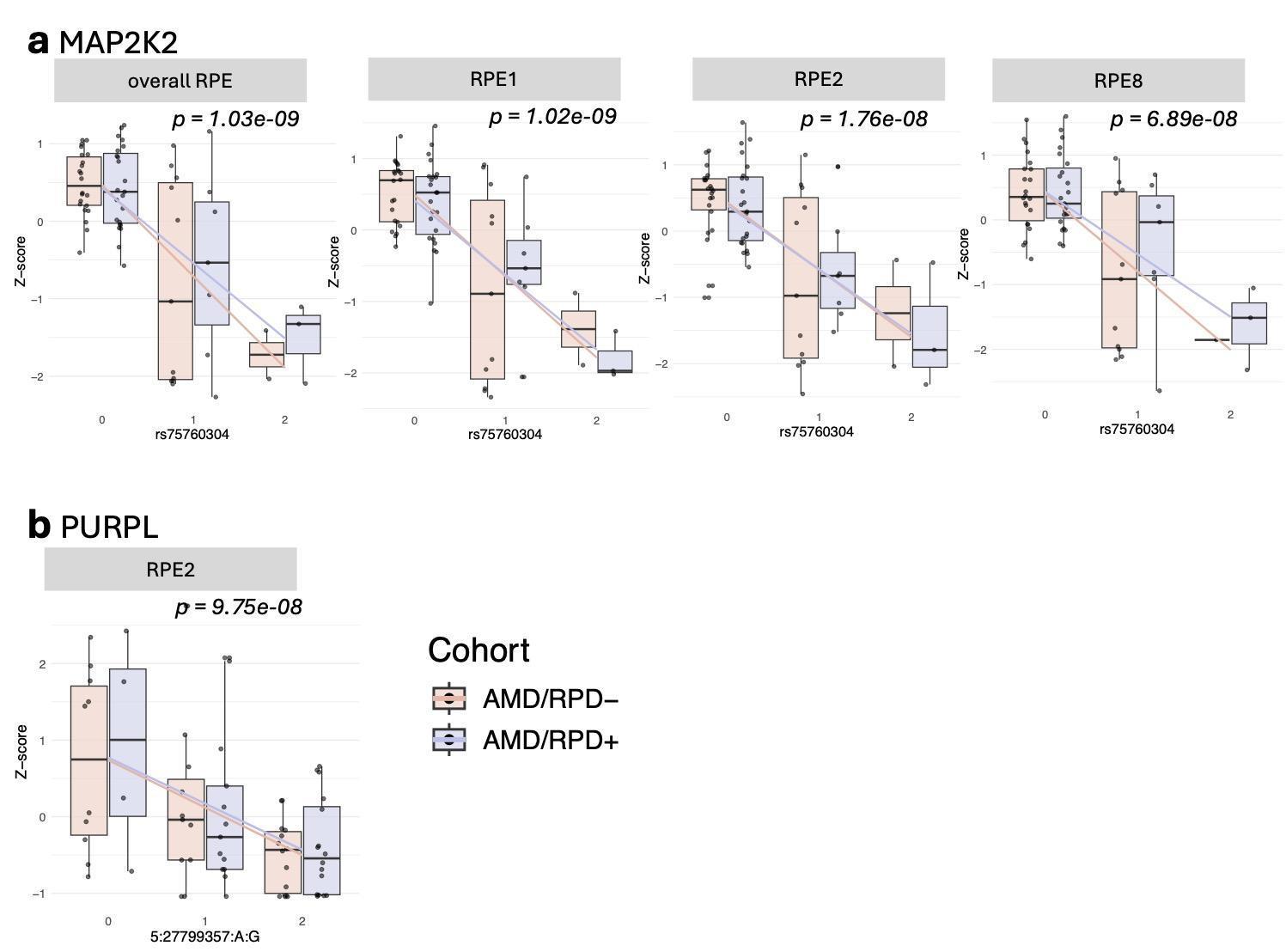
**

**Fig S4.** **Examples of eQTLs in RPE subpopulations.** (**a**) Expression of *MAP2K2* stratified by rs75760304 genotype in overall RPE and in individual subpopulations (RPE1, RPE2, RPE8), shown separately for AMD/RPD- and AMD/RPD+ cohorts. (**b**) Expression of *PURPL* stratified by 5:27799357:A:G genotype in RPE2, shown separately for AMD/RPD- and AMD/RPD+ cohorts. Expression values are represented as z-scores.


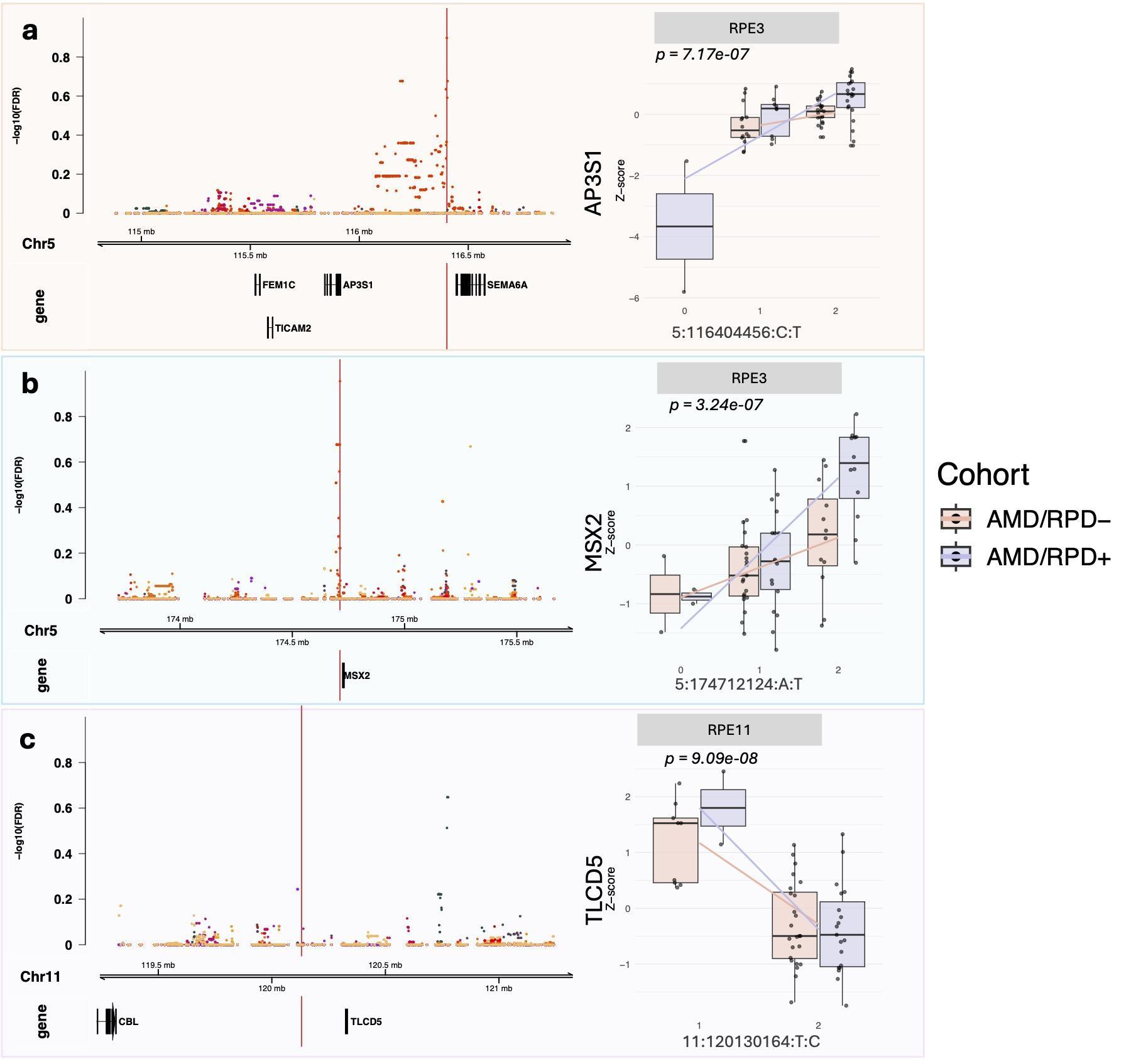
**Fig S5. Examples of disease-interacting eQTLs in RPE subpopulations.** Regional association plots (left) depict local association signals (-log10 false discovery rate) across the genomic locus, with the lead SNP indicated by a vertical red line. Boxplots (right) show expression stratified by genotype and cohort, with regression lines illustrating allele dosage effects. (**a**) *AP3S1* in RPE3 (5:116404456 C>T; *p* = 7.17 × 10⁻⁷). (**b**) *MSX2* in RPE3 (5:174712124 A>T; *p* = 3.24 × 10⁻⁷). (**c**) *TLCD5* in RPE11 (11:120130164 T>C; *p* = 9.09 × 10⁻⁸).


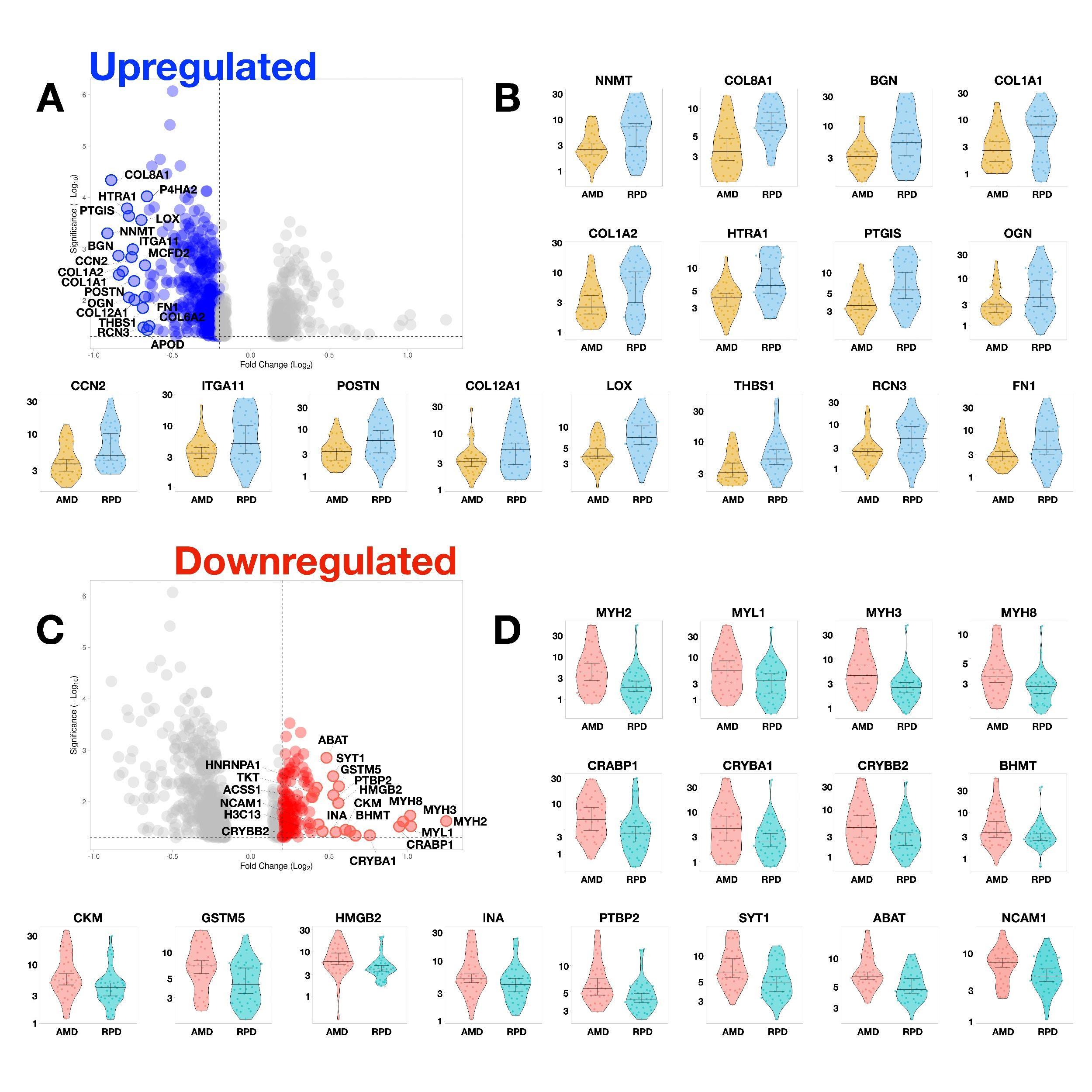


**Fig S6. Top differentially expressed proteins between the two cohorts.** Violin plots showing the top 16 proteins most significantly up- or down-regulated in AMD/RPD+ relative to AMD/RPD- RPE cells based on log₂-normalised TMT proteomic data. Each violin represents the distribution of protein abundance.


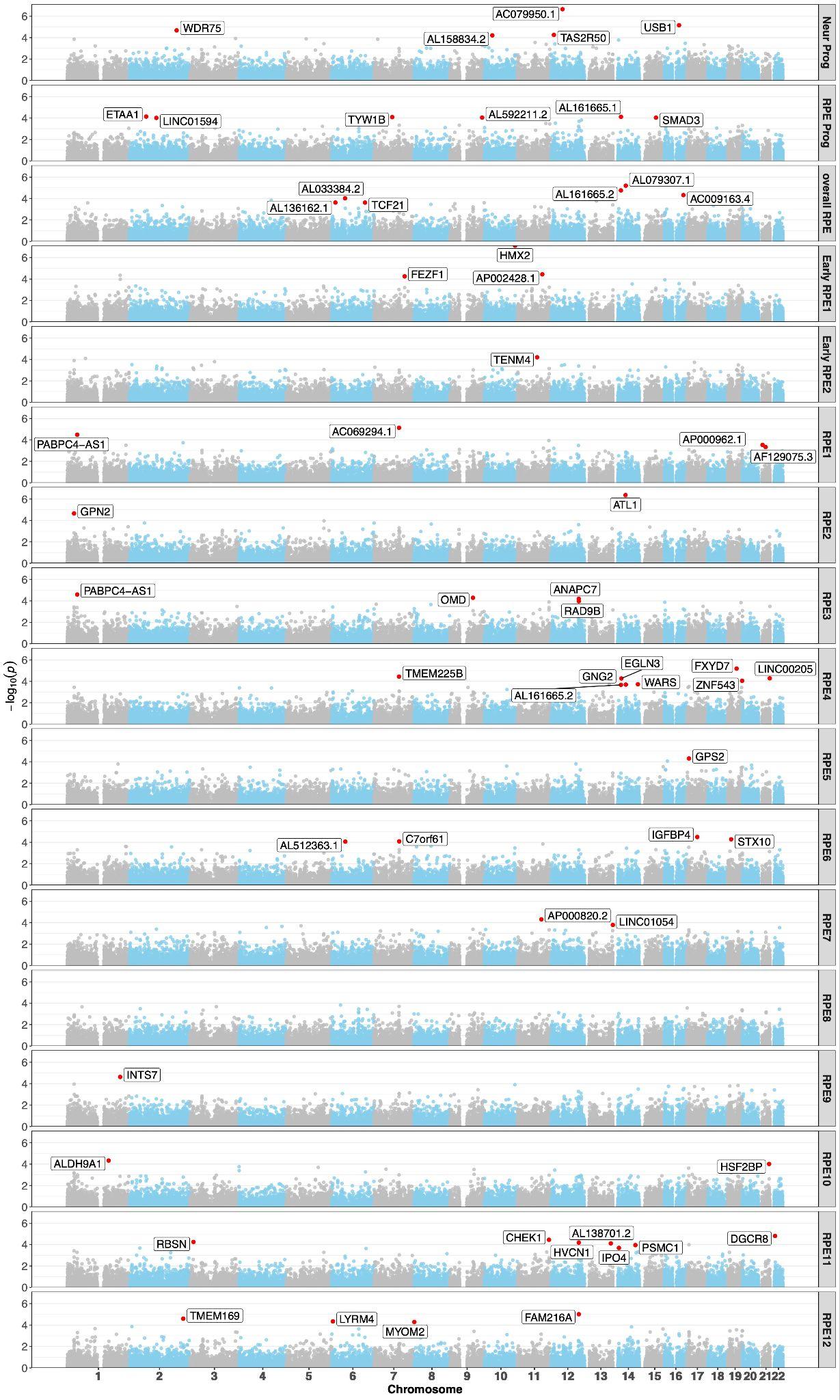


**Fig S7. Transcriptome-wide association study (TWAS) of RPD risk across RPE populations.** Manhattan plots showing TWAS results for overall and subpopulation-specific RPE transcriptomic models. Each point represents a gene-level association between genetically predicted expression and RPD risk, plotted by genomic position. The red dashed line denotes the significance threshold (false discovery rate < 0.1). Significant loci are labelled.

**
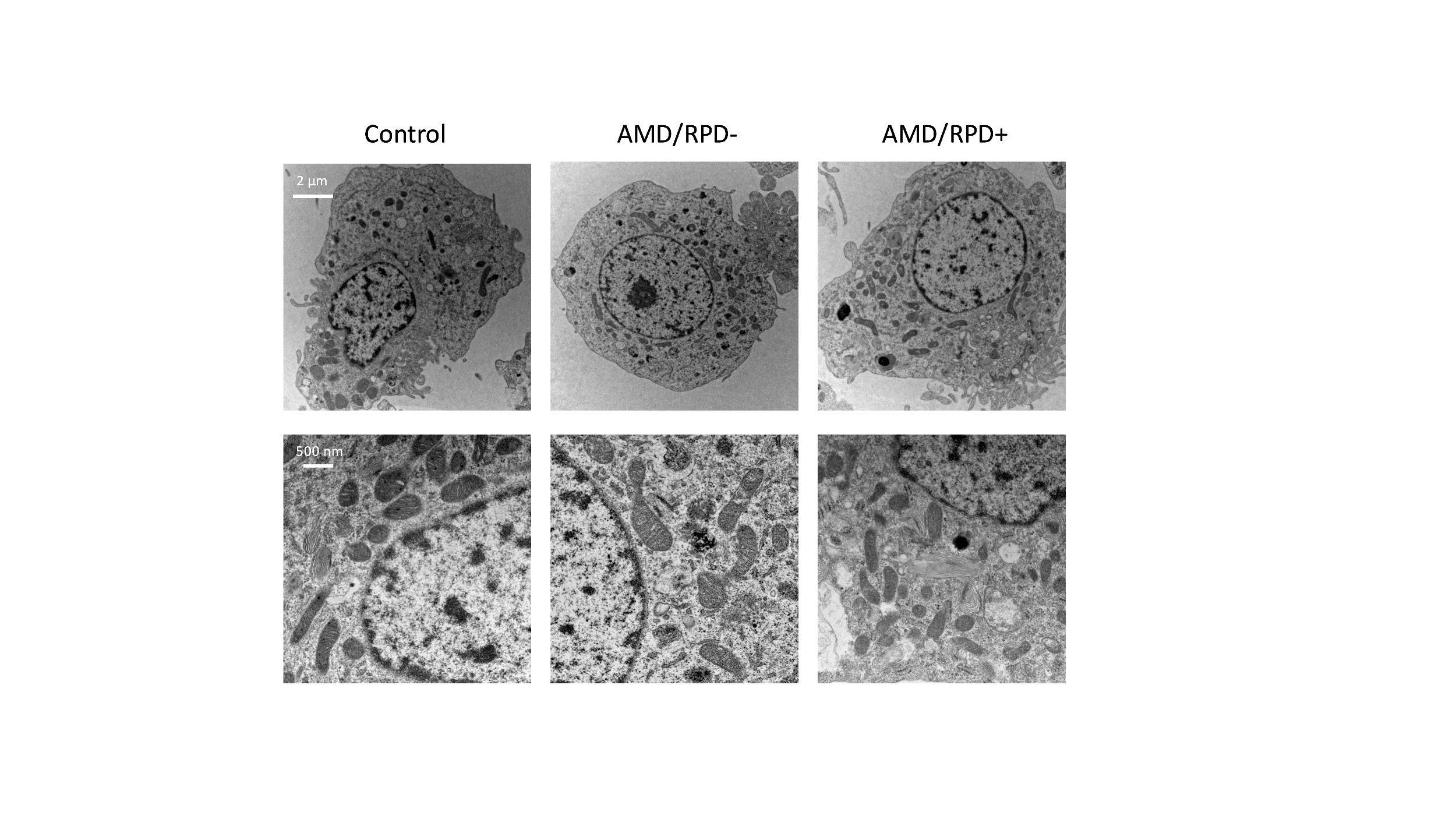
**

**Fig S8. Transmission electron microscopy of iPSC-derived RPE cells.** Representative images of RPE cells derived from control, AMD/RPD-, and AMD/RPD+ donors. Top row: low-magnification views showing overall cell morphology, including nuclei, cytoplasmic organisation, and organelles (scale bar: 2 µm). Bottom row: higher-magnification views highlighting mitochondrial ultrastructure and cytoplasmic detail (scale bar: 500 nm). No consistent ultrastructural differences were observed between cohorts.

**
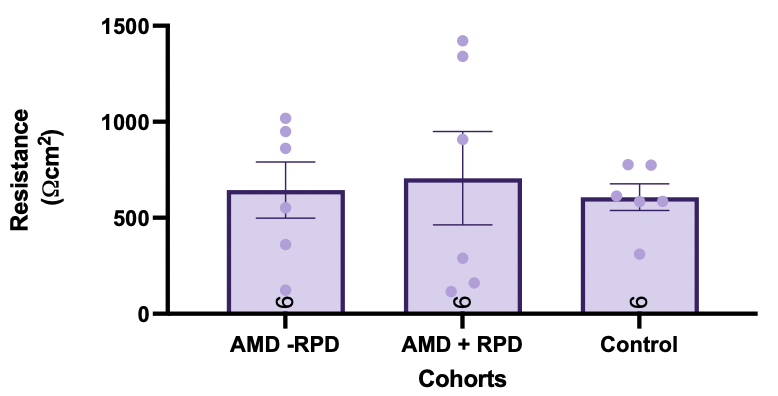
**

**Fig S9. Transepithelial electrical resistance (TEER) in iPSC-derived RPE cells**. Resistance values are from control, AMD/RPD- (AMD - RPD), AMD/RPD+ (AMD + RPD) and control lines (n= 6 lines per cohort). Each dot represents the average of technical triplicates for each iPSC-derived line. Data are shown as mean ± SD, with statistical significance determined by one way ANOVA (p < 0.05).


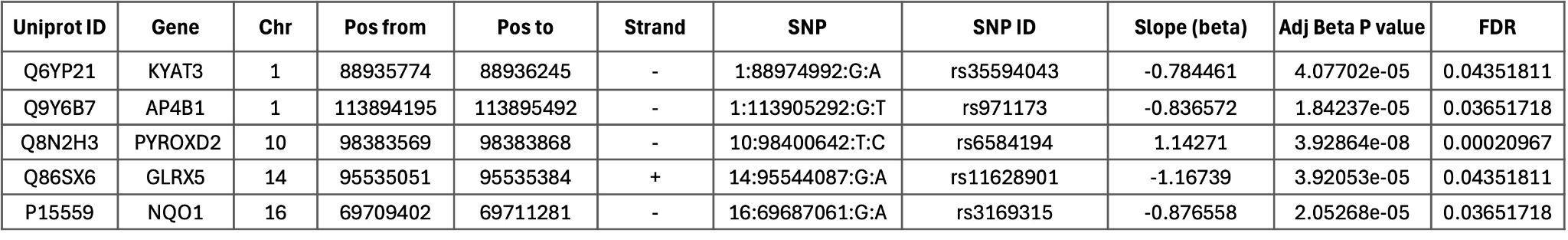


**Table S1.** **Full details of significant pQTLs.** Complete information including genomic coordinates and alleles.
